# Supplementary material for: How do project managers’ competencies impact project success? A systematic literature review
Source: PLoS One. 2023 Dec 7;18(12):e0295417. doi: 10.1371/journal.pone.0295417 (PMC10703200; doi:10.1371/journal.pone.0295417)
Supplement: S1 Table — Notes: PMG = Project manager, PS = Project success. (PDF) [file pone.0295417.s002.pdf]

S1 Table. Inclusion and exclusion criteria used in the SLR.

Criteria	Inclusion	Exclusion
**Type of document**	Peer-reviewed scholarly research articles.	Systematic literature reviews, review papers, conference proceedings, books, book chapters, and dissertations.
**Publication timeline**	January 2010 and October 2022.	Before January 2010 or after October 2022.
**Language**	English.	Other languages.
**Type of study**	Quantitative approach measuring PMGs' competencies as independent variable and PS as a dependent variable.	Quantitative - descriptive studies, systematic literature reviews, meta-analysis, qualitative studies, study cases.
**Population**	PMGs or similar positions (e.g., project director, project leader, senior PMG, department manager, functional manager, team leader).	Positions different from PMGs.
**Context**	Professional settings.	Research conducted in non-professional settings (e.g., students or graduates in high school, university projects or projects related to project-based learning).

Notes: PMG = Project manager; PS = Project success.
