# Supplementary material for: How do project managers’ competencies impact project success? A systematic literature review
Source: PLoS One. 2023 Dec 7;18(12):e0295417. doi: 10.1371/journal.pone.0295417 (PMC10703200; doi:10.1371/journal.pone.0295417)
Supplement: S2 Table — Notes: PMG = Project manager, PS = Project success. (PDF) [file pone.0295417.s003.pdf]

S2 Table. Quality assessment criteria scoring guide.

Criteria	Description	Points	Criteria
**QC1. Research questions**	Research questions, objectives and/or hypotheses have been appropriately established.	1	Completely clear and related to the title of the article.
		0.5	Somehow clear and related to the title of the article.
		0	Not reported or unclear.
**QC2. Study design**	The study design is well described and appropriate for answering the research questions.	1	Described in detail and completely appropriate.
		0.5	Partially described and somehow appropriate.
		0	Not described, or poorly described.
**QC3. Sample representativeness**	The sample and population of the study are clearly described, and its size is sufficient to carry out the proposed analysis.	1	Well described and truly representative of the target population (all subjects or random sampling).
		0.5	Well described but it is non-representative of the average in the target population (non-random sampling).
		0	No description of the sampling strategy.
**QC4. Response rate**	The response rate is reported and above 50%.	1	Participation/response rate was reported, and it was at least 50%.
		0.5	Participation/response rate was reported, but it was less than 50%.
		0	Not reported.
**QC5. PMG's competencies measurement**	The instruments for measuring PMG's competencies are well described and design based.	1	PMGs' competencies are well operationalized and measured with reliable, valid, and previously piloted instruments.
		0.5	PMGs' competencies are operationalized and measured but reliability and source of the instrument are not reported.
		0	No description of the measurement tool.
**QC6. PS measurement**	The instruments for measuring project success are well described and design based.	1	PS is well operationalized and measured with reliable, valid, and previously piloted instruments.
		0.5	PS is well operationalized and measured but reliability and source of the instrument are not reported.
		0	No description of the measurement tool.

Criteria	Description	Points	Criteria
**QC7. Statistical analysis**	Statistical method was appropriate for the question being answered and sufficiently described to enable its replication.	1	The statistical test used to analyze the data is clearly described and appropriate.
		0.5	The statistical test used to analyze the data is clearly described but not completely appropriate.
		0	No clear description of the statistical method used.
**QC8. Results**	The research questions were adequately answered.	1	Results completely allow to answer the research questions, hypothesis, or objectives of the study.
		0.5	Results partially allow to answer the research questions, hypothesis, or objectives of the study.
		0	Results do not allow to answer the research question, hypothesis, or objectives of the study.
**QC9. Statistical significance**	Statistical significance of associations was tested and reported.	1	The measurement of the association is presented, including confidence intervals and the probability level (*p* value).
		0.5	Only confidence intervals or the probability level (*p* value) for the measurement of the association is presented.
		0	Not reported.
**QC10. Conclusions**	The conclusions are clearly described and based on the results.	1	Conclusions are clear and derived from the results of the study.
		0.5	Conclusions are somehow clear and somehow derived from the results of the study.
		0	Not reported.

Notes: PMG = Project manager; PS = Project success.
