# Supplementary material for: How do project managers’ competencies impact project success? A systematic literature review
Source: PLoS One. 2023 Dec 7;18(12):e0295417. doi: 10.1371/journal.pone.0295417 (PMC10703200; doi:10.1371/journal.pone.0295417)
Supplement: S3 Table — Notes: QC1 = Research questions; QC2 = Study design; QC3 = Sample representativeness; QC4 = Response rate; QC5 = PMG’s competencies measurement; QC6 = PS measurement; QC7 = Statistical analysis; QC8 = Results; QC9 = Statistical significance; QC10 = Conclusions; SLR = Systematic literature review. (PDF) [file pone.0295417.s004.pdf]

S3 Table. Quality assessment results.

Author	Reviewer	Quality criteria										Total	Conclusion
		QC1	QC2	QC3	QC4	QC5	QC6	QC7	QC8	QC9	QC10		
Afzal et al. (2018)	R1	1	1	0.5	0	0.5	0.5	0.5	1	0.5	1	6.5	Not included in the SLR
	R2	1	1	0.5	0	0.5	0.5	0.5	1	0.5	1	6.5	Not included in the SLR
Ahmed and Lodhi (2021)	R1	1	1	0.5	1	1	1	1	1	0.5	1	9	Included in the SLR
	R2	1	0.5	0.5	1	1	1	0.5	1	0.5	0.5	7.5	Included in the SLR
Elmezain et al. (2021)	R1	1	1	0.5	1	1	1	1	1	0.5	1	9	Included in the SLR
	R2	1	0.5	1	1	1	1	0.5	1	0.5	1	8.5	Included in the SLR
Irfan et al. (2021)	R1	1	1	0.5	1	1	1	1	1	0.5	1	9	Included in the SLR
	R2	1	0.5	1	1	1	1	0.5	1	0.5	1	8.5	Included in the SLR
Khan et al. (2020)	R1	1	1	0.5	0	1	1	1	1	0.5	1	8	Included in the SLR
	R2	1	1	0.5	0.5	1	1	1	1	0.5	1	8.5	Included in the SLR
Lima and Quevedo-Silva (2020)	R1	1	1	0.5	0	1	1	1	1	0.5	1	8	Included in the SLR
	R2	1	1	0.5	0	1	1	0.5	1	0.5	1	7.5	Included in the SLR
Maqbool et al. (2017)	R1	1	1	1	1	1	1	1	1	0.5	1	9.5	Included in the SLR
	R2	1	1	1	1	1	1	0.5	1	0.5	1	9	Included in the SLR
Müller and Turner (2010)	R1	1	1	0.5	0	1	1	1	1	0.5	1	8	Included in the SLR
	R2	1	1	1	1	1	1	1	1	0.5	1	9.5	Included in the SLR
Murali and Venkatesh (2019)	R1	1	0	0.5	0	0	0	0.5	1	0.5	1	4.5	Not included in the SLR
	R2	1	0	0.5	0	0	0	0.5	1	0.5	1	4.5	Not included in the SLR
Ozorhon et al. (2022)	R1	1	0.5	0.5	0.5	0.5	0.5	1	1	0	1	6.5	Not included in the SLR
	R2	1	0.5	0.5	0.5	0	0	1	1	0.5	1	6	Not included in the SLR

Author	Reviewer	Quality criteria										Total	Conclusion
		QC1	QC2	QC3	QC4	QC5	QC6	QC7	QC8	QC9	QC10		
Podgórska and Pichlak (2019)	R1	1	1	0.5	1	1	1	1	1	0.5	1	9	Included in the SLR
	R2	1	1	1	1	0.5	0.5	1	1	0.5	1	8.5	Included in the SLR
Rana and Shuja (2022)	R1	1	1	0.5	0	1	1	1	1	0.5	1	8	Included in the SLR
	R2	1	0.5	0.5	0	1	1	1	1	0.5	1	7.5	Included in the SLR
Sampaio et al. (2022)	R1	1	1	0.5	1	1	1	1	1	0.5	1	9	Included in the SLR
	R2	1	1	0.5	1	1	1	1	1	0.5	1	9	Included in the SLR

Notes: QC1 = Research questions; QC2 = Study design; QC3 = Sample representativeness; QC4 = Response rate; QC5 = PMG's competencies measurement; QC6 = PS measurement; QC7 = Statistical analysis; QC8 = Results; QC9 = Statistical significance; QC10 = Conclusions; SLR = Systematic literature review.
