# Supplementary material for: How do project managers’ competencies impact project success? A systematic literature review
Source: PLoS One. 2023 Dec 7;18(12):e0295417. doi: 10.1371/journal.pone.0295417 (PMC10703200; doi:10.1371/journal.pone.0295417)
Supplement: S4 Table — (PDF) [file pone.0295417.s005.pdf]

S4 Table. Brief description of the Project Managers' competencies in included articles.

Dimension	Competencies	Definition	References
Cognitive	Creativity	The ability to be imaginative and innovative to solve problems, face challenges, envision the future and generate novel ideas and methods.	Müller and Turner (2010), Ochoa et al. (2018), Podgórska and Pichlak (2019), Sampaio et al. (2022).
	Decision-making	The ability to make decisions based on rational and intuitive perceptions after gathering relevant information from different sources.	Müller and Turner (2010), Ochoa et al. (2018), Podgórska and Pichlak (2019).
	Strategic perspective	The ability to be aware of broader issues and implications, balance short- and long-term considerations, and identify opportunities or threats.	Podgórska and Pichlak (2019), Müller and Turner (2010).
Personal	Conscientiousness	The ability to display a clear commitment to a course of action when faced with a challenge and to match "words and needs" when encouraging others to support the chosen direction.	Podgórska and Pichlak (2019), Müller and Turner (2010).
	Emotional intelligence	The ability to recognize, understand, express, manage, and be aware of both one's own emotions and the emotions of others.	Podgórska and Pichlak (2019), Sampaio et al. (2022).
	Results orientation	The ability to show determination and commitment to achieve specific objectives and outcomes.	Podgórska and Pichlak (2019), Müller and Turner (2010).
Social	Communication	The ability to communicate effectively, provide effective feedback, and engage others by conveying verbal and non-verbal messages encompassing relevant information, knowledge, and emotions that impact the audience.	Müller and Turner (2010), Ochoa et al. (2018), Podgórska and Pichlak (2019), Sampaio et al. (2022).
	Conflict management	The ability to promote a constructive dialogue and problem resolution during conflict situations.	Caldas et al. (2016).
	Interpersonal relations	The ability to establish, maintain, and articulate relationships with others and, when necessary, change their viewpoint by understanding team members' positions and recognizing their own needs and perspectives to achieve cooperation among all team members.	Lima-Quevedo et al. (2020), Ochoa et al. (2018), Podgórska and Pichlak (2019), Müller and Turner (2010).
	Leadership	The ability to lead a project team toward accomplishing project goals, empower team members, and boost the development of others' competencies.	Sampaio et al. (2022).
	Teamwork	The ability to collaborate with others and integrate and achieve synergy among the team members.	Lima-Quevedo et al. (2020), Ochoa et al. (2018).
Sustainability	Ethics	The ability to perform based on principles and values of justice, transparency, honesty, and integrity.	Ochoa et al. (2018), Sampaio et al. (2022).
