# Supplementary material for: How do project managers’ competencies impact project success? A systematic literature review
Source: PLoS One. 2023 Dec 7;18(12):e0295417. doi: 10.1371/journal.pone.0295417 (PMC10703200; doi:10.1371/journal.pone.0295417)
Supplement: S5 Table — (PDF) [file pone.0295417.s006.pdf]

S5 Table. Brief description of the project success criteria in included articles.

Project success dimensions	Success criteria	Description	References
Impact on stakeholders	Clients' satisfaction	The level of satisfaction of the clients with the project deliverables and outcomes.	Imam and Zaheer (2021), Santos et al. (2020).
	Users' satisfaction	The final users' satisfaction level with the project deliverables and outcomes.	Maqbool et al. (2017).
	Providers' satisfaction	The level of satisfaction among the suppliers involved in the project regarding the project management processes.	Maqbool et al. (2017).
	Teams' satisfaction	The level of satisfaction of the project team members with the overall project management and performance.	Maqbool et al. (2017).
	Other stakeholders' satisfaction	The level of satisfaction and perceived advantages of stakeholders over short, medium, or long-term periods derived from the project's outcomes.	Aga et al. (2016), Maqbool et al. (2017), Santos et al. (2020).
Impact on organization	Visible short-term improvements	The benefits derived by the organization or business within a one- to two-year span following project completion.	Ika and Pinto (2022), Imam and Zaheer (2021).
	Long-term improvements	The sustained positive results achieved by the organization four or more years after the project completion.	Ika and Pinto (2022), Imam and Zaheer (2021).
General project management	Project efficiency	The fulfillment of project completion within the estimated schedule, budget, and in alignment with required quality standards.	Santos et al. (2020).
	Achieving purpose and objectives	The achievement of the project's purpose and objectives.	Müller and Turner (2010).
	Project self-defined criteria	Other relevant criteria depending on the type of project or industry in which it was developed, or specific criteria defined by the stakeholders involved in the project.	Maqbool et al. (2017).
