# Supplementary material for: How do project managers’ competencies impact project success? A systematic literature review
Source: PLoS One. 2023 Dec 7;18(12):e0295417. doi: 10.1371/journal.pone.0295417 (PMC10703200; doi:10.1371/journal.pone.0295417)
Supplement: Attachment — Submitted filename: Response to Reviewer 2.pdf [file pone.0295417.s008.pdf]

Response to Reviewer 2 Comments

We thank you for your time reviewing our article and the valuable comments that helped improve its quality. We have welcomed your observations. Below, you will find the details of the changes made. These changes are highlighted in blue the document.

Point 1: Abstract – Which dimensions? It will be better if mentioned here.

> **Response 1:** Following your recommendation, we mentioned the three dimensions of competencies (sustainability, cultural and digital) which we were referring to in the original version of the abstract.

Point 2: Line 3 – Project management and project managers should be explained more here. By writing a paragraph per each, you can explain what it is, how it is done, and what features it requires.

> **Response 2:** As you requested, we incorporated two introductory paragraphs in Section 2 (lines 54–66) to define project management as a discipline and the role of project managers.

Point 3: Line 41 – Dimensions should be mentioned here and explained by writing one sentence.

> **Response 3:** We indicated the four resulting dimensions that were used to categorize PMGs' competencies (lines 42 and 43).

Point 4: Line 50 – Project managers' competencies and project success.

> **Response 4:** Following your suggestion, we modified this heading (line 52).

Point 5: Line 52-53 – These questions should be explained to readers: What is the project? What does it consist of? Whose responsibilities are more important? What do Project managers do? What does project success mean?

> **Response 5:** We incorporated a brief definition of projects and mentioned some responsibilities of PMGs in lines 56–59 and 63–66, respectively. The definition of PS is shown in lines 144–147.

Point 6: Line 55 – PMGs' Competencies

> **Response 6:** Following your suggestion, we modified the heading of Section 2.1. (line 75).

Point 7: Line 123-125 – So, Does the PS not include time, cost, quality, management practices, stakeholders expectations? I believe it consists of all. You should explain that PS covers project efficiency and project performance.

Response 7: Regarding your comment, PS includes traditional performance (also called efficiency) criteria such as time, cost, and quality, as well as other criteria such as stakeholders' satisfaction, impact on organizations, among others. We modified the statement presented in lines 144 and 145 to ensure clarity.

Point 8: Line 129 – SLR method should be explained more. Who prefers this method and why? What is its reliability and validity? How to do it?

Response 8: Following your request, we incorporated a paragraph in Section 3 regarding the SLR method (lines 151–157).

Point 9: Line 135 – You should explain why you picked these databases. Because of their …

Response 9: We added the rationale for choosing the databases in lines 167–168.

Point 10: Line 188 – Have you mentioned the results of these literature reviews in this paper?

Response 10: These three articles were removed from the SLR because they did not meet the inclusion criteria regarding the type of document. In addition, these three articles were not related to the research questions proposed in our study.

Point 11: Line 280 – Figure 1 does not show square and circle nodes.

Response 11: The figure display error is due to the format in which the file was sent. Since the image is in EPS and PDF format (both suitable for use at the time of production of the article) and converted to Word format, it is likely that some graphic element has been misaligned. We recommend viewing the image directly from the platform.

Point 12: Table 2, Line 313 – Competencies should be explained in the paper. Readers should understand what is their importance.

Response 12: Following your recommendation, we incorporated a supplementary table (S4 Table) where we present a definition of each competence. This table is mentioned in Table 2 (line 322).

Point 13: Table 3, Line 333 – Success criterias should be explained in the paper. Readers should understand what is their importance.

Response 13: As you suggested, we present a definition of each success criteria in a supplementary table (S5 Table). This table is mentioned in Table 3 (line 342).

Point 14: Line 337 – Statistical results should be given here and table 4. R value, significance value etc.

Response 14: Following your request, we mentioned the statistical results of each included articled in Table 4 and throughout the text in Section 4.4.

Point 15: Lines 353, 370, 384 – Statistical values should be given in brackets for all results when you give citations, if there.

Response 15: Request answered in Point 14.

Point 16: Line 431 – Using original resources is better.

Response 16: Following your suggestion, we removed the phrase "who cited Sunindijo et al. [81] as their framework source".

Point 17: Line 522 – According to these conclusions, what are your suggestions? What should PMGs do to project success? Which competencies should be developed?

Response 17: Following your request, we incorporated a paragraph in Section 7 (Conclusions) regarding three recommendations for PMGs (lines 585–593).

Additional changes were incorporated in the manuscript to meet all reviewer's recommendations. A summary of these modifications is present below:

- Section 3 (Method) was modified by optimizing the paragraphs related to inclusion and exclusion criteria (lines 197 and 198), quality assessment criteria (line 231), and the thematic analysis (lines 250–259). The detailed information regarding inclusion and exclusion criteria, quality assessment criteria, and quality assessment results can be found in supplementary materials (S1 Table, S2 Table, and S3 Table).

- A paragraph regarding the psychosocial perspective incorporated throughout the study and its relevance for the study of the relationship between project managers' competencies and project success (lines 544–557).
