# Supplementary material for: How do project managers’ competencies impact project success? A systematic literature review
Source: PLoS One. 2023 Dec 7;18(12):e0295417. doi: 10.1371/journal.pone.0295417 (PMC10703200; doi:10.1371/journal.pone.0295417)
Supplement: Attachment — Submitted filename: PONE-D-23-22151_reviewer.docx [file pone.0295417.s007.docx]

PLOS ONE

**How do project managers’ competencies impact project success? A systematic literature review**

--Manuscript Draft--

| **Manuscript Number:** | PONE-D-23-22151 |
| --- | --- |
| **Article Type:** | Research Article |
| **Full Title:** | How do project managers’ competencies impact project success? A systematic literature review |
| **Short Title:** | How do project managers’ competencies impact project success? |
| **Corresponding Author:** | Paola Ochoa  Escuela Superior Politécnica del Litoral Guayaquil, Guayas ECUADOR |
| **Keywords:** | project manager; competencies; project success; Systematic literature review |
| **Abstract:** | Despite the existence of systematic literature reviews focused on examining the factors contributing to project success, there remains a scarcity of reviews addressing the relationship between the project managers’ competencies and project success. To fill this gap in the literature, this review aimed to evaluate peer-reviewed articles, published between 2010 and 2022, and analyze the impact of project managers’ competencies on project success. The Web of Science, Scopus, ScienceDirect, and ProQuest electronic databases were first consulted in September 2021, with an update in August and October 2022. A total of 232 titles were analyzed. Ten articles met the criteria and were fully reviewed. A content analysis and a citation network were carried out to analyze the included articles. The analysis revealed that the existing literature has primarily explored the influence of competencies from the personal and social dimensions, such as leadership, communication, and emotional intelligence, on project success. Conversely, competencies from other dimensions have received less attention in the literature. In addition, this review contributes to the literature by providing a holistic categorization of competencies associated with project success and examining and organizing project success criteria into three dimensions. |
| **Order of Authors:** | Paola Ochoa Pacheco |
|  | David Coello-Montecel |
|  | Michelle Tello |
|  | Virginia Lasio |
|  | Alfredo Armijos |
| **Additional Information:** |  |
| **Question** | **Response** |
| **Financial Disclosure**  Enter a financial disclosure statement that describes the sources of funding for the work included in this submission. Review the [submission guidelines](http://journals.plos.org/plosone/s/submission-guidelines#loc-financial-disclosure-statement) for detailed requirements. View published research articles from [*PLOS ONE*](http://journals.plos.org/plosone/) for specific examples.  This statement is required for submission and **will appear in the published article** if the submission is accepted. Please make sure it is accurate. | This study did not receive any specific grant from funding agencies in the public, commercial, or not-for-profit sectors. |

| **Unfunded studies**  Enter: *The author(s) received no specific funding for this work.*  **Funded studies**  Enter a statement with the following details:   - Initials of the authors who received each award - Grant numbers awarded to each author - The full name of each funder - URL of each funder website - Did the sponsors or funders play any role in the study design, data collection and analysis, decision to publish, or preparation of the manuscript? - **NO** - Include this sentence at the end of your statement: *The funders had no role in study design, data collection and analysis, decision to publish, or preparation of the manuscript.* - **YES** - Specify the role(s) played. |  |
| --- | --- |
| * typeset |  |
| **Competing Interests**  Use the instructions below to enter a competing interest statement for this submission. On behalf of all authors, disclose any [competing interests](http://journals.plos.org/plosone/s/competing-interests) that could be perceived to bias this work—acknowledging all financial support and any other relevant financial or non- financial competing interests.  This statement is required for submission and **will appear in the published article** if the submission is accepted. Please make sure it is accurate and that any funding sources listed in your Funding Information later in the submission form are also declared in your Financial Disclosure statement.  View published research articles from  [*PLOS ONE*](http://journals.plos.org/plosone/) for specific examples. | The authors declare no conflict of interest. |

| **NO authors have competing interests**  Enter: *The authors have declared that no competing interests exist.*  **Authors with competing interests**  Enter competing interest details beginning with this statement:  *I have read the journal's policy and the authors of this manuscript have the following competing interests: [insert competing interests here]* |  |
| --- | --- |
| * typeset |  |
| **Ethics Statement**  Enter an ethics statement for this submission. This statement is required if the study involved:   - Human participants - Human specimens or tissue - Vertebrate animals or cephalopods - Vertebrate embryos or tissues - Field research   Write "N/A" if the submission does not require an ethics statement.  General guidance is provided below. Consult the [submission guidelines](http://journals.plos.org/plosone/s/submission-guidelines#loc-guidelines-for-specific-study-types) for detailed instructions. **Make sure that all information entered here is included in the Methods section of the manuscript.** | Not applicable. |

| **Format for specific study types**  **Human Subject Research (involving human participants and/or tissue)**   - Give the name of the institutional review board or ethics committee that approved the study - Include the approval number and/or a statement indicating approval of this research - Indicate the form of consent obtained (written/oral) or the reason that consent was not obtained (e.g. the data were analyzed anonymously)   **Animal Research (involving vertebrate animals, embryos or tissues)**   - Provide the name of the Institutional Animal Care and Use Committee (IACUC) or other relevant ethics board that reviewed the study protocol, and indicate whether they approved this research or granted a formal waiver of ethical approval - Include an approval number if one was obtained - If the study involved *non-human primates*, add *additional details* about animal welfare and steps taken to ameliorate suffering - If anesthesia, euthanasia, or any kind of animal sacrifice is part of the study, include briefly which substances and/or methods were applied   **Field Research**  Include the following details if this study involves the collection of plant, animal, or other materials from a natural setting:   - Field permit number - Name of the institution or relevant body that granted permission |  |
| --- | --- |
|  |  |
| **Data Availability**  Authors are required to make all data underlying the findings described fully available, without restriction, and from the time of publication. PLOS allows rare exceptions to address legal and ethical concerns. See the [PLOS Data Policy](http://journals.plos.org/plosone/s/data-availability) and [FAQ](http://journals.plos.org/plosone/s/data-availability#loc-faqs-for-data-policy) for detailed information. | Yes - all data are fully available without restriction |

| A Data Availability Statement describing where the data can be found is required at submission. Your answers to this question constitute the Data Availability Statement and **will be published in the article**, if accepted. |  |
| --- | --- |
| **Important:** Stating ‘data available on request from the author’ is not sufficient. If your data are only available upon request, select ‘No’ for the first question and explain your exceptional situation in the text box. |  |
| Do the authors confirm that all data underlying the findings described in their manuscript are fully available without restriction? |  |
| **Describe where the data may be found in full sentences. If you are copying our sample text, replace any instances of XXX with the appropriate details.**   - If the data are **held or will be held in a public repository**, include URLs, accession numbers or DOIs. If this information will only be available after acceptance, indicate this by ticking the box below. For example: *All XXX files are available from the XXX database (accession number(s) XXX, XXX.*). - If the data are all contained **within the manuscript and/or Supporting Information files**, enter the following: *All relevant data are within the manuscript and its Supporting Information files.* - If neither of these applies but you are able to provide **details of access elsewhere**, with or without limitations, please do so. For example:   *Data cannot be shared publicly because of [XXX]. Data are available from the XXX Institutional Data Access / Ethics Committee (contact via XXX) for researchers who meet the criteria for access to confidential data.*  *The data underlying the results presented in the study are available from (include the name of the third party* | All files are available either within the manuscript (Tables 1, 2, 3, 4) or as supplementary files. Hyperlinks are provided within the manuscript after the reference list. |

| *and contact information or URL).*   - This text is appropriate if the data are owned by a third party and authors do not have permission to share the data.   * typeset |  |
| --- | --- |
| Additional data availability information: | Tick here if the URLs/accession numbers/DOIs will be available only after acceptance of the manuscript for publication so that we can ensure their inclusion before publication.; Tick here if your circumstances are not covered by the questions above and you need the journal’s help to make your data available. |

Manuscript	[Click here to](https://www.editorialmanager.com/pone/download.aspx?id=33739429&guid=567575c5-b0f9-40aa-9476-08dc5d066190&scheme=1)

[access/download;Manuscript;manuscript_SLR_PMGcompetenci](https://www.editorialmanager.com/pone/download.aspx?id=33739429&guid=567575c5-b0f9-40aa-9476-08dc5d066190&scheme=1)

### How do project managers’ competencies impact project success? A systematic literature review

**Paola Ochoa Pacheco**

ESPAE Graduate School of Management, Escuela Superior Politécnica del Litoral (ESPOL), Ecuador ORCID: <https://orcid.org/0000-0001-6657-6406>

Email: [pjochoa@espol.edu.ec](mailto:pjochoa@espol.edu.ec)

### David Coello-Montecel

ESPAE Graduate School of Management, Escuela Superior Politécnica del Litoral (ESPOL), Ecuador ORCID: <https://orcid.org/0000-0001-8183-7344>

Email: [dacoello@espol.edu.ec](mailto:dacoello@espol.edu.ec)

### Michelle Tello

ESPAE Graduate School of Management, Escuela Superior Politécnica del Litoral (ESPOL), Ecuador ORCID: <https://orcid.org/0000-0002-7449-5654>

Email: [mgtello@espol.edu.ec](mailto:mgtello@espol.edu.ec)

### Virginia Lasio

ESPAE Graduate School of Management, Escuela Superior Politécnica del Litoral (ESPOL), Ecuador Email: [mlasio@espol.edu.ec](mailto:mlasio@espol.edu.ec)

### Alfredo Armijos

ESPAE Graduate School of Management, Escuela Superior Politécnica del Litoral (ESPOL), Ecuador Email: [alarmijo@espol.edu.ec](mailto:alarmijo@espol.edu.ec)

**Corresponding autor:**

Paola Ochoa

Email: [pjochoa@espol.edu.ec](mailto:pjochoa@espol.edu.ec)

ESPAE Graduate School of Management ESPOL Campus Las Peñas, Malecón 100 y Loja Guayaquil – Ecuador

Phone: (+593) 4 208 1084 Ext. 125

### How do project managers’ competencies impact project success? A systematic literature review

**Abstract**

Despite the existence of systematic literature reviews focused on examining the factors contributing to project success, there remains a scarcity of reviews addressing the relationship between the project managers’ competencies and project success. To fill this gap in the literature, this review aimed to evaluate peer-reviewed articles, published between 2010 and 2022, and analyze the impact of project managers’ competencies on project success. The Web of Science, Scopus, ScienceDirect, and ProQuest electronic databases were first consulted in September 2021, with an update in August and October 2022. A total of 232 titles were analyzed. Ten articles met the criteria and were fully reviewed. A content analysis and a citation network were carried out to analyze the included articles. The analysis revealed that the existing literature has primarily explored the influence of competencies from the personal and social dimensions, such as leadership, communication, and emotional intelligence, on project success. Conversely, competencies from other dimensions have received less attention in the literature. In addition, this review contributes to the literature by providing a holistic categorization of competencies associated with project success and examining and organizing project success criteria into three dimensions.

**Keywords:** project manager, competencies, project success, systematic literature review

### 1 How do project managers’ competencies impact project success? A systematic literature review

2

### 3 1. Introduction

4

5 The profound economic, technological, and social changes experienced in recent years [1,2] have
6 compelled organizations to devise strategies and implement initiatives to adapt to uncertain environments
7 [3]. Projects allow organizations to face these challenges by leveraging their expertise and capabilities to
8 deliver solutions aligned with business objectives [4]. Project management (PM) has been acknowledged as
9 a valuable discipline for managers and professionals implementing strategic organizational transformations
10 [1]. Given the shortage of qualified talent to execute strategic initiatives and drive change [5], the project
11 managers’ (PMGs) competencies have garnered significant attention from scholars [6–11] and PM
12 institutions [12,13]. Consequently, a substantial body of literature has devoted considerable effort to
13 delineating the competencies that have the potential to enhance projects’ positive outcomes [14–18].

14

15 There has been a growing interest in exploring the individual and organizational factors contributing
16 to project success (PS). At the individual level, the PMGs’ leadership style [19], job satisfaction [20], trust
17 [21], job crafting [22], and work-family conflict [23], among other factors have been associated with PS. At
18 the organizational level, scholars have highlighted that PS can be influenced by innovative climate [24],
19 organizational culture [25], cultural diversity [26], governance [27], knowledge sharing and perceived trust
20 and cohesion of the team [28,29], among others.

21

22 Despite the existence of systematic literature reviews (SLRs) that summarize the available evidence
23 regarding factors associated with PS [30], there remains a scarcity of SLRs focusing on PMGs’
24 competencies [31,32] and their impact on PS. Only a limited number of SLRs [33] have been dedicated to
25 identifying the competencies essential for achieving PS. However, to the best of our knowledge, an SLR
26 focused on analyzing the relationship between PMGs’ competencies and PS has not been conducted before.
27 To fill this gap in the literature, this SLR analyzes the existing evidence regarding the relationship between
28 PMGs’ competencies and PS. Therefore, the present SLR was designed to address the following research
29 questions: (RQ1) Which PMGs’ competencies are the most examined in the existing literature? (RQ2)
30 Which success criteria are the most considered when measuring PS in the existing literature? (RQ3) Which
31 PMGs’ competencies have a relationship with PS?

32

33 This SLR contributes to the literature on the PM discipline in four ways. Firstly, it fills a gap in the
34 existing literature by employing the SLR methodology to comprehensively synthesize the available
35 evidence from published empirical studies concerning the relationship between PMGs’ competencies and
36 PS. Secondly, it employs a thematic analysis and a holistic perspective to categorize the PMGs’
37 competencies associated with PS. This methodological approach provides a comprehensive framework for
38 understanding the diverse competencies relevant to PS. Thirdly, it offers an insightful analysis of a graphical
39 representation that showcases the primary authors and institutions that have significantly influenced the
40 conceptualization of PMGs’ competencies. Lastly, it examines the criteria utilized for measuring PS in the
41 included articles and organizes them into three dimensions, enhancing the understanding of the multifaceted
42 nature of PS assessment. By addressing these aspects, this SLR contributes to advancing knowledge in PM.

43

44 The subsequent sections of this paper are structured as follows. Section 2 presents the conceptualization of
45 PMGs’ competencies and PS. Section 3 outlines the procedure for conducting the SLR, encompassing the
46 search strategy, study selection, data extraction, and analysis. The findings derived from the SLR are
47 presented in Section 4. Lastly, the paper concludes by discussing the implications of the results, highlighting
48 the strengths and limitations of the SLR, and offering final remarks.

49

### 50 2. Competencies and project success

51

52 This section provides an overview of the conceptualizations of competencies adopted in the PM literature,
53 and briefly discuss the evolution of the PS dimensions.

54

55	2.1. Competencies

56

57 Various conceptualizations of competencies have been explored in the existing literature [16,34–38].
58 Within the PM discipline, several studies [18,39–42] have aligned with the classical definition proposed by
59 Boyatzis [35]. According to his framework, competencies encompass the underlying characteristics of an
60 individual, including knowledge, skills, abilities, attitudes, and more, that collectively enable the
61 achievement of high performance. These elements have served as a foundational basis for scholars [9,43,44]
62 and institutions [12,13], who have further expanded the scope to develop frameworks tailored explicitly to
63 the domain of PM.

64

65 PM institutions, including the Project Management Institute (PMI) and the International Project
66 Management Association (IPMA), have played a crucial role in the definition and development of various
67 standards and frameworks that pertain to the competencies of PMGs [45]. Several studies [16,46,47] have
68 employed these institutional standards to define competencies. The subsequent section provides a concise
69 overview of these institutional frameworks.

70

71 According to the IPMA [13], competencies comprise the practical application of knowledges, skills, and
72 abilities to achieve desired outcomes. This framework recognizes the interconnectedness of these elements,
73 as proficiency entails acquiring relevant knowledge and developing skills that, when put into practice,
74 enable professionals to manage projects effectively and successfully. Similarly, the PMI [12] defines
75 competencies as the capability to carry out activities within a portfolio, program, or project setting that yield
76 anticipated results based on established and accepted standards. This definition builds upon Boyatzis’ [35]
77 elements and aligns with the IPMA [13] perspective, but it also emphasizes compliance by acknowledging
78 the significance of adhering to current regulations and guidelines to meet stakeholders’ expectations. More
79 recently, the PMI [48] introduced the concept of *power skills*, which refers to the abilities and behaviors that
80 facilitate working with others and enable project professionals to succeed in the workplace, align projects
81 to organizational objectives, and motivate teams to contribute value to the organization and its customers.

82

83 The scholarly literature [8,37,43,44] has significantly contributed to the conceptualization of the
84 competencies required by PMGs by incorporating key elements from the PM discipline. For instance, Hanna
85 et al. [43] emphasized the evolving nature of projects. They argued that competencies entail the
86 demonstrated ability to perform project activities within a dynamic environment, leading to expected
87 outcomes based on established standards. Building upon this perspective, Bashir et al. [44] defined
88 competencies as a meta-ability that integrates skills, aptitudes, and abilities to perform throughout the
89 project life cycle, from initiation to closing, intending to achieve expected results. Moreover, Crawford [49]
90 posited a close relationship between PMGs’ competencies and PS. Recent literature has underscored the
91 pivotal role of PMGs’ competencies in attaining higher levels of success, enhancing efficiency and
92 effectiveness, and consequently increasing the likelihood of PS [8].

93

94	2.2 Project success

95

96 This section provides an overview of the historical development of the conceptualization of PS, tracing
97 its progression from a unidimensional to a more comprehensive and multidimensional concept [50]. It also
98 aims to identify the dimensions and criteria incorporated into the concept in recent years. Furthermore, it
99 defines PS and examines its distinctions from related concepts, such as project performance and efficiency.
100 100
101 Traditionally, scholars [39,51–53] have viewed PS as a combination of success factors and criteria. On
102 the one hand, success factors refer to the significant elements that enhance the probability of achieving
103 success. On the other hand, success criteria comprise a set of measures used to evaluate if the project can
104 be judged as successful [39]. This SLR specifically focuses on PS criteria.
105 105
106 The measurement criteria for assessing PS have undergone significant evolution to encompass the
107 complex and dynamic nature of projects, resulting in the development of more comprehensive models
108 [52,54]. Initially, PS frameworks primarily focused on efficiency criteria, commonly referred to as the
109 “golden triangle,” “iron triangle,” or “holy trinity,” which encompassed elements such as time, cost, and
110 quality [54]. Subsequent models expanded to incorporate dimensions of client and project team satisfaction
111 [55]. From the year 2000 onwards, the emergence of integrative models took into account additional
112 dimensions, including realized benefits to the business or organization [56,57], satisfaction levels of internal
113 and external stakeholders such as end-users, suppliers, and other relevant parties [58], the impacts on the
114 community and environment [59], long-term effects like the creation of new markets or product lines
115 [56,60], and investment returns [61].
116 116
117 The conceptual boundaries between PS, project performance, project efficiency, and PM success have often
118 been blurred. On the one hand, PM success represents a conventional measure of PS that primarily focuses
119 on time, cost, and quality, assessed upon project completion [62,63]. These criteria are also called project
120 efficiency [64]. On the other hand, project performance refers to the degree to which management practices
121 and processes contribute to the achievement of goals and objectives, as well as the fulfillment of
122 stakeholders’ expectations. It is typically evaluated throughout project execution and upon completion
123 [54,65]. In contrast, PS represents a broader and multidimensional concept encompassing the achievement
124 of goals and objectives determined by key stakeholders after project completion [63,64], as well as the long-
125 term impacts of the project [66].
126 126

### 127 3. Method

128 128
129 The SLR was undertaken to investigate the abovementioned research questions and followed the guidelines
130 outlined in the Preferred Reporting Items for Systematic Reviews and Meta-Analyses (PRISMA). The
131 protocol employed for conducting this SLR is elaborated upon in the following section.
132 132
133 3.1. Search strategy
134 134
135 The Web of Science, Scopus, ScienceDirect, and ProQuest electronic databases were selected for this SLR.
136 The databases were first consulted in September 2021, with an update in August and October 2022, by
137 searching the following keywords in the title of the article: “competence,” “competency,” “competences,”
138 “competencies,” “skill,” “skills,” and “project success,” without any additional constraint. The search was
139 performed by two of the authors using the following search strings:
140 140
141 •	*Scopus database:* TITLE ((competence) OR (competency) OR (competences) OR (competencies)
142 OR (skill) OR (skills)) AND TITLE ((project AND success))
143 •	*Web of Science database:* TI=(competence OR competency OR competences OR competencies OR
144 skill OR skills) AND TI=(project success)
145 •	*ScienceDirect database:* Title: (competence OR competency OR competences OR competencies
146 OR skill OR skills) AND (project success)
147 •	*ProQuest database:* title((competence OR competency OR competences OR competencies OR skill
148 OR skills)) AND title((project success))
149 149
150 The metadata of the records (title, authors, document type, source title, author keywords, abstract,
151 publication year, volume number, issue number, and DOI) was exported, compared, and saved on Microsoft
152 Excel spreadsheets to remove duplicated studies and conduct the screening process.
153 153
154 3.2. Study selection
155 155
156 The study selection process comprised several stages to find relevant articles for the review. The initial
157 research resulted in 232 articles. After removing duplicated records, 172 articles were considered for the
158 next stages. The procedures followed by the authors are described below.
159 159
160 *3.2.1. Inclusion and exclusion criteria*
161 161
162 The inclusion and exclusion criteria for document selection in this review were based on various factors,
163 including publication timeline, document type, language, study type, population, and context. To be
164 included in this review, documents had to meet the following criteria: (1) they had to be peer-reviewed
165 scholarly research articles, (2) they had to be published between January 2010 and October 2022, (3) they
166 had to be written in English, (4) they had to have a quantitative approach measuring PMG’s competencies
167 as independent variable and PS as a dependent variable, (5) the study population had to consist of PMGs or
168 similar positions (e.g., project director, project leader, senior PMG, department manager, functional
169 manager, team leader), and (6) the research work had to be conducted in professional settings. The study
170 selection process did not impose restrictions on industry, project type, or project size to ensure a broader
171 scope and encompass various perspectives. This approach allowed for the retrieval of peer-reviewed
172 scholarly articles that addressed the research questions of this SLR. Initially, 172 articles were evaluated,
173 and after applying the inclusion criteria, 131 records were removed. Subsequently, 41 research articles
174 remained for the screening process.
175 175
176 *3.2.2. Article screening process*
177 177
178 After applying inclusion and exclusion criteria, the retained articles were screened by title, abstract, and full
179 text. This process was conducted by two of the authors independently. The reasons for excluding articles
180 were reported in each step. The exclusion criteria were objectively applied. Studies were excluded if the
181 relationship between PMGs’ competencies and PS was not examined. Each reviewer’s number and list of
182 excluded articles were compared after the screening. In those cases where there was disagreement between
183 reviewers, a third author reviewed the article and discussed it with the other two authors to reach a
184 consensus. Eligible articles were included in the final review. Ineligible articles were formally excluded,
185 with the reasons for exclusion noted.
186 186
187 Out of 41 articles, seven were excluded based on the title. In this step, the main reasons for exclusion were:
188 (a) the study was related to project-based learning (*n* = 4), (b) the article was a literature review (*n* = 3), and
189 (c) the article was a case of study (*n* = 1). The retained 34 articles were screened by abstract. After analyzing
190 the abstract of each article, eight were removed because of the following: (a) the study had a qualitative
191 design (*n* = 1), (b) the article was a case of study (*n* = 1), (c) the article analyzed only leadership styles (*n* =
192 1), (d) the article was theoretical (*n* = 2), (e) the study was not conducted in a PM professional setting (*n* =
193 1), and (f) the article did not analyze the relationship between PMGs’ competencies and PS (*n* = 2). Finally,
194 the full-text screening was carried out on 26 articles. Thirteen records were excluded based on the following
195 reasons: (a) PMGs’ competencies were not measured (*n* = 3), (b) the article was theoretical (*n* = 3), and (c)
196 the study did not analyze the relationship between PMGs’ competencies and PS (*n* = 7). After the whole
197 screening process, 13 articles were considered for quality assessment.
198 198
199 *3.2.3. Quality assessment*
200 200
201 The quality assessment focused on ten quality criteria statements: (1) The research questions, objectives, or
202 hypothesis were appropriately established; (2) The study design was well described and appropriate for
203 answering the research questions; (3) The sample and population of the study were clearly described, and
204 its size was sufficient to carry out the proposed analysis; (4) The response rate was reported and above 50%;
205 (5) The instruments used for measuring PMGs’ competencies were well described and design-based; (6)
206 The instrument used for measuring PS was well described and design-based; (7) The statistical method was
207 appropriate and sufficiently described to enable them to be repeated; (8) The research questions were
208 adequately answered; (9) The statistical significance of associations was tested and reported; (10) The
209 conclusions were clearly described and based on the results.
210 210
211 The abovementioned criteria were adapted from the Newcastle-Ottawa Quality Assessment Scale (adapted
212 for cross-sectional studies), the Appraisal Tool for Cross-Sectional Studies (AXIS), and similar studies [67].
213 Each statement had three rating options coded as “Yes” (1 point), “No” (0 points), and “Partial” (0.5 points).
214 Articles with a score of 7.5 points or higher were included in the final sample. The quality assessment was
215 carried out by two authors independently. The results were compared, and the differences found were
216 discussed to make a final decision. In this phase, three articles were removed. Ten articles were selected to
217 conduct the analysis and answer the research questions of this SLR. Figure 1 summarizes the data extraction
218 procedure through a PRISMA flow.
219 219
220 *Figure 1 about here*
221 **Figure 1.** PRISMA flow chart for systematic literature review.
222 222
223 3.3. Data extraction and analysis
224 224
225 Three authors analyzed the articles for data extraction, including sample characteristics, country, setting,
226 independent and outcome variable(s), data analysis procedures, and main findings. These data were
227 synthesized in Table 4.
228 228
229 A thematic analysis was conducted to identify the dimensions of PMGs’ competencies and PS criteria used
230 in the included articles, following the procedures proposed by Nowell et al. [68]:
231 231
232 (i)	*Familiarization with the data.* The authors read and analyzed the content of each article.
233 (ii)	*Generation of initial code*s. Each author generated a list of competencies and PS criteria extracted
234 from each article. The resulting lists were compared and matched to get a final version.
235 (iii)	*Creation of themes*. Categories were created by grouping similar competencies and PS criteria.
236 Each of the authors carried out this process individually. The resulting lists were compared and
237 matched to get a final version as in the previous step.
238 (iv)	*Definition and naming of themes*. Once the final list of competencies and PS were obtained and
239 the main categories were defined, each category was named based on theoretical foundations. This
240 process was carried out jointly by the three authors.
241 241
242 When studying topics such as PMGs’ competencies, an important issue is how authors support their choice
243 regarding what competencies to include in their work. This decision is important since it shapes the structure
244 of the research field. Thus, a citation network analysis (CNA) was carried out to map the structure of the
245 PMGs’ competencies research field. In CNA, research documents serve as nodes, and the connections
246 between them are represented by citations [69]. CNA is a practical approach for identifying contributions
247 to a specific topic and uncovering relationships within the scholarly literature, thereby revealing patterns of
248 influence and collaboration [70]. In this SLR, the ten included articles relied on citations of prior works to
249 select the pertinent PMGs’ competencies. These cited references were used to build a network representing
250 the relevant frameworks in the included articles. The citation network was generated using the visNetwork
251 package in RStudio.
252 252

### 253 4. Results

254 254
255 4.1. Study characteristics
256 256
257 The main characteristics of the articles included in this SLR are shown in Table 1. Data were collected from
258 11 countries across five regions: Asia, Europe, North America, Oceania, and South America. Notably,
259 Pakistan emerged as the most prolific country, with five papers published between 2010 and 2022, followed
260 by the USA (*n* = 2) and Brazil (*n* = 2). Most studies were published within the last five years (*n* = 9). Out
261 of the ten articles, eight were published in journals categorized in the Q1 (*n* = 5) and Q2 (*n* = 3) impact
262 quartiles. In terms of study design, most articles employed a purely quantitative approach (*n* = 8), while two
263 utilized mixed methods. For instance, Sampaio et al. [71] conducted a systematic review to identify the
264 competencies to be included in their subsequent questionnaire, while Podgórska and Pichlak [72] employed
265 a mixed-method approach comprising semi-structured interviews and a survey questionnaire.
266 266
267 **Table 1.** Selected publications for analysis.

**Year	No. of published**

**articles**

**Country/Region	Journal	Journal Impact Quartile**

**Article included in the SLR**

2022	2	South America (Brazil),

International Journal of

Q1	Sampaio et al. [71]

North America (Canada and Managing Projects in USA), Asia (China), Europe Business

(Ireland and Portugal)

|  | | Pakistan | Pakistan Journal of Commerce and Social Science | Q1 | Rana and Shuja [73] |
| --- | --- | --- | --- | --- | --- |
| 2021 | 3 | Egypt | Brazilian Journal of Operations and Production Management | Q2 | Elmezain et al. [74] |
|  |  | Pakistan | International Journal of Information Technology Project Management | Q2 | Ahmed and Lodhi [75] |
|  |  | Pakistan | Sustainability | Q1 | Irfan et al. [76] |
| 2020 | 2 | Brazil | International Journal of Project Organisation and Management | Q3 | Lima and Quevedo- Silva [77] |
|  |  | Pakistan | Pacific Business Review International | N/A | Khan et al. [78] |
| 2019 | 1 | Poland | International Journal of Managing Projects in Business | Q1 | Podgórska and Pichlak [72] |
| 2017 | 1 | Pakistan | Project Management Journal | Q1 | Maqbool et al. [15] |
| 2010 | 1 | Europe, North America, Australia, New Zealand, among others | Baltic Journal of Management | Q2 | Müller & Turner [39] |

Note: The Journal Impact Quartile was based on Scopus CiteScore. N/A = Journal Impact Quartile not available.

268 268
269 269
270 4.2. Project managers’ competencies
271 271
272 *4.2.1 The most influential theoretical frameworks*
273 273
274 The majority of articles (*n* = 9) included in the SLR employed an existing framework to identify the PMGs’
275 competencies that were examined in their empirical analyses. However, in the study conducted by Sampaio
276 et al. [71], a comprehensive literature review was undertaken to determine the specific competencies that
277 should be considered for testing their impact on PS.
278 278
279 A CNA was conducted to explore the interrelationships among the ten articles included in this SLR and to
280 identify the most influential frameworks for defining and determining the PMGs’ competencies. Figure 1
281 visually represents the articles included in the SLR as square nodes and the studies that have contributed to
282 conceptualizing PMGs’ competencies as circle nodes. The size of each node reflects the number of citations
283 it has received. The diagram layout was arranged such that the most frequently cited studies are positioned
284 in the center, while less frequently cited ones are placed towards the periphery. A summary of the most
285 influential works in PMGs’ competencies is provided below.
286 286
287 Four articles included in this SLR [39,72,73,75] employed a common framework developed by Dulewicz
288 and Higgs [79]. This framework encompasses 15 leadership competencies categorized into three
289 dimensions: intellectual competencies (critical analysis and judgment, vision and imagination, strategic
290 perspective), managerial competencies (managing resources, engaging communication, empowering,
291 developing, achieving), and emotional competencies (self-awareness, emotional resilience, intuitiveness,
292 interpersonal sensitivity, influence, motivation, conscientiousness). Additionally, two articles [15,77] drew
293 upon Clarke’s [80] study, which identified four main PMGs’ competencies: communication, teamwork,
294 attentiveness, and managing conflict. Other frameworks utilized in the SLR articles were proposed by
295 Sunindijo [81], Katz [82], Nguyen and Hadikusumo [83], and Ofori [84]. These frameworks shared common
296 elements, emphasizing the significance of communication, leadership, managing emotions, and
297 interpersonal relationships as essential competencies for PMGs. Notably, the Project Manager Competency
298 Development Framework [12] and the Individual Competence Baseline for Project Management [13] were
299 among the most cited institutional frameworks employed in the SLR articles.
300 300
301 *Figure 2 about here*
302 **Figure 2.** The PMGs’ competencies research network from a citation network perspective.
303 Notes. Square nodes represent the articles in the SLR (*n* = 10), while circle nodes denote the studies that contributed
304 to conceptualizing PMGs’ competencies. The number of citations gives the size of the node.
305 305
306 *4.2.2 Categorization of project managers’ competencies*
307 307
308 Several common competencies were identified based on the review of competencies reported in each article.
309 These competencies were categorized into four dimensions based in previous studies [11,31,85], as
310 presented in Table 2: cognitive, personal, social, and sustainability. It should be noted that not all
311 competencies were consistently referred to by the same name across the included articles. Therefore, the
312 names used to denote a specific competence in each article are listed in the third column of Table 2.
313 **Table 2.** PMGs’ competencies in the articles included in the SLR (*n* = 10).

**Authors**

**Dimensions	Competencies	Associated terms**

**Ahmed & Lodhi [75] Elmezain et al. [74] Irfan et al. [76] Khan et al. [78]**

**Lima & Quevedo-Silva [77] Maqbool et al. [15]**

**Müller and Turner [39] Podgórska and Pichlak [72] Rana and Shuja [73]**

**Sampaio et al. [71]**

| Cognitive | Creativity | Creativity, vision and imagination, resourcefulness, creative thinking, imagination |  |  |  |  |  |  | X | X | X | X |
| --- | --- | --- | --- | --- | --- | --- | --- | --- | --- | --- | --- | --- |
|  | Decision-making | Decision-making skills, intuitiveness, critical analysis, and judgment |  |  | X |  |  |  | X | X | X |  |
|  | Strategic perspective | Strategic perspective |  |  |  |  |  |  | X | X | X | X |
| Personal | Conscientiousness | Conscientiousness |  |  |  |  |  |  | X | X | X |  |
|  | Emotional intelligence | Emotional intelligence, self-awareness, resilience, stress management, self-control, sensitivity | X | X |  |  | X | X | X | X | X | X |
|  | Results orientation | Results orientation, motivation, achieving |  |  |  |  |  |  | X | X | X | X |
| Social | Communication | Engaging communication, communication management, communication, verbal skills, feedback |  |  |  | X | X | X | X | X | X | X |
|  | Conflict management | Managing conflict, resolving conflicts |  |  | X |  | X | X |  |  |  |  |
|  | Interpersonal relations | Attentiveness, interpersonal linkages, interpersonal skills, influence |  | X | X | X | X |  | X | X |  |  |
|  | Leadership | Political skills, transformational leadership, empowering, developing, coaching |  | X | X |  |  | X | X | X | X | X |
|  | Teamwork | Team building, teamwork |  |  | X |  | X | X |  |  |  |  |
| Sustainability | Ethics | Transparency, honesty, integrity |  | X |  |  |  |  |  |  |  | X |
| 314 |  |  |  |  |  |  |  |  |  |  |  |  |

315	4.3. Project success criteria

316

317 Previous literature has traditionally focused on PS measures related to cost, time, and quality. However, the
318 findings of the SLR indicate a growing tendency to incorporate a broader range of success criteria. Table 3
319 presents a categorization of the different success criteria reported in the included articles.
320 320
321 The first dimension pertains to the impact on stakeholders, encompassing clients, users, providers, the
322 project team, and other relevant parties. While stakeholder impact is commonly assessed through
323 satisfaction measures, some studies consider alternative indicators such as the acceptability of the product,
324 perceived benefits [73], or the fulfillment of stakeholder expectations [74]. Less frequently addressed are
325 measures related to the impact on the organization, for which two criteria were identified: (i) visible short-
326 term improvements in organizational outcomes or performance [73,75,76], and (ii) long-term
327 improvements, such as the development of new technologies or the initiation of future projects [39,72,75].
328 Additional criteria related to the project management process were identified, encompassing project
329 performance, achievement of the project’s primary objectives, other self-defined criteria related to project
330 management, and compliance with procedures, safety regulations, and environmental standards. Project
331 performance indicators include the traditional metrics of cost, time, and quality of the project’s deliverables

332	[39,72].

333	**Table 3.** Project criteria success in included articles.

**Articles included in the SLR**

**Project success dimensions**

**Success criteria**

**Ahmed & Lodhi [75]**

**Elmezain et al. [74]**

**Irfan et al. [76]**

**Lima & Quevedo-Silva [77]**

**Maqbool et al. [15]**

**Müller and Turner [39]**

**Podgórska and Pichlak [72]**

**Rana and Shuja [73]**

**Sampaio et al. [71]**

|  | Impact on | Clients satisfaction | X | X |  |  |  | X | X | X |  |
| --- | --- | --- | --- | --- | --- | --- | --- | --- | --- | --- | --- |
|  | stakeholders | Users satisfaction |  |  | X |  | X | X | X | X | X |
|  |  | Providers satisfaction |  |  |  |  | X | X | X |  |  |
|  |  | Teams satisfaction | X |  |  |  | X | X | X | X |  |
|  |  | Other stakeholders satisfaction |  |  |  |  | X | X | X |  |  |
|  | Impact on | Visible short-term improvements | X |  | X |  |  |  |  | X |  |
|  | organization | Long-term improvements | X |  |  |  |  | X | X |  |  |
|  | General project | Project efficiency (time, cost, and quality) | X | X | X | X | X | X | X | X | X |
|  | management | Achieving purpose and objectives |  |  | X | X | X | X | X | X | X |
|  |  | Project self-defined criteria |  |  |  |  | X | X | X |  |  |
|  |  | Compliance with safety and environmental procedures and regulations |  |  | X |  |  |  |  |  |  |
| 334 |  |  |  |  |  |  |  |  |  |  |  |

335	4.4. Empirical analysis of the relationship between project managers’ competencies and project success

336

337 Table 4 presents a comprehensive overview of the research methods and results employed in the included
338 studies. Several studies conducted correlational analyses to examine the relationship between competencies
339 and various PS criteria [71,72,75], as well as overall PS [15]. Regression analysis was a common method
340 to assess the predictive impact of PMGs’ competencies on PS criteria in the selected articles [39,72,74,75].
341 Additionally, some studies [73,74,76,78] employed structural equation modeling (SEM) or partial least
342 squares (PLS) to analyze the predictive effect of PMGs’ competencies, modeled as second-order constructs,
343 on PS.
344 344
345 *4.4.1. Relationship between cognitive competencies and project success*
346 346
347 Cognitive competencies encompassed creativity, decision-making, and strategic perspective. Findings from
348 two studies revealed a positive correlation between creativity and various PS criteria, such as accomplishing
349 project objectives, project efficiency, user satisfaction [71], and suppliers’ satisfaction [72]. While creativity
350 significantly predicted project efficiency, its effect on achieving project objectives and user satisfaction was
351 not statistically significant [71]. Two studies included in the SLR [39,72] provided evidence concerning the
352 relationship between strategic perspective and PS. Firstly, Müller and Turner [39] found that this
353 competence influences project efficiency and self-defined success criteria. Secondly, Podgórska & Pichlak
354 [72] reported that strategic perspective is significantly associated with all the analyzed PS criteria except for
355 project efficiency and self-defined success criteria. Regarding the decision-making competence, Müller and
356 Turner [39] did not identify any significant predictive effects of this competence. However, Podgórska and
357 Pichlak [72] observed significant positive correlations between decision-making and all the PS criteria, with
358 the highest coefficients observed for self-defined success criteria, end-user satisfaction, and satisfaction of
359 other stakeholders.
360 360
361 *4.4.2. Relationship between personal competencies and project success*
362 362
363 Personal competencies included emotional intelligence, results orientation, and conscientiousness. Among
364 these competencies, emotional intelligence has received significant attention in the included studies. Out of
365 the ten studies, eight explored the relationship between emotional intelligence and PS. The evidence
366 revealed direct and significant predictive effects of emotional intelligence on various PS criteria, such as
367 end-user satisfaction [71], achievement of project objectives [39], and overall PS [15].
368 368
369 Regarding results orientation, Sampaio et al. [71] demonstrated that this competence had a predictive effect
370 on project efficiency. Correlational analysis revealed a higher correlation between this competence with PS
371 criteria related to user satisfaction [71] and satisfaction of other stakeholders [72]. The conscientiousness
372 competence was examined in the studies conducted by Müller and Turner [39] and Podgórska and Pichlak
373 [72]. This competence emerged as a significant predictor of team satisfaction and the achievement of project
374 objectives [39]. Furthermore, the effects of conscientiousness could vary depending on the type and
375 complexity of the project [72].
376 376
377 *4.4.3. Relationship between social competencies and project success*
378 378
379 Based on the articles included in this review, social competencies, such as communication, leadership,
380 interpersonal relations, conflict management, and teamwork, tend to be associated with PS. Leadership has
381 been extensively studied in the project management literature and was addressed in seven out of the ten
382 articles included in this review. Correlational analysis revealed that leadership shows significant
383 associations with nearly all PS criteria, being its highest correlation with the user satisfaction criterion
384 [71,72]. Regarding its predictive effect on PS, Sampaio et al. [71] reported a non-significant effect of this
385 competence on some criteria, such as achieving project’s purpose, project efficiency, and stakeholders’
386 satisfaction, while other studies found a significant effect on team satisfaction criterion [39] and an overall
387 PS measure [15,74].
388 388
389 Regarding communication, correlational analysis showed that this competence is highly correlated with
390 stakeholders’ satisfaction criterion [71,72]. Maqbool et al. [15] found that communication had the strongest
391 correlation with a general measure of PS among all competencies included in their study. The predictive
392 effect of this competence on PS was confirmed by Lima and Quevedo-Silva [77], Khan et al. [78] and
393 Podgórska and Pichlak [72]. However, Sampaio et al. [71] and Müller and Turner [39] reported non-
394 significant effects of communication of PS criteria.
395 395
396 Interpersonal relations showed significant positive associations with PS criteria, with the strongest
397 coefficients on achieving project’s purpose [72]. Müller and Turner [39] found that this competence has a
398 significant predictive effect on other stakeholders’ satisfaction criteria, while two studies [77,78] reported
399 its predictive effect on a general PS measure. Finally, significant positive associations of conflict
400 management and teamwork with PS were reported by Maqbool et al. [15]. However, its predictive effect on
401 individual PS criteria were not estimated on any of the included articles.
402 402
403 *4.4.4. Relationship between sustainability competencies and project success*
404 404
405 According to Elmezain et al. [74], the capacity to demonstrate integrity, sincerity, and authenticity, and to
406 inspire confidence and trust in others, is relevant for achieving PS. The authors emphasized that PMGs who
407 possess integrity play a crucial role in the advancement of any project. Similarly, Sampaio et al. [71]
408 highlighted ethics, conceptualized as transparency, integrity, and honesty, as the most significant
409 competence for achieving PS in terms of goal attainment.
410 **Table 4.** Summary of included articles.

| **No.** | **Author** | **Sample** | **Sector** | **Response rate** | **Analysis tool** | **Main results** |
| --- | --- | --- | --- | --- | --- | --- |
| 1 | Sampaio et al. [71] | *Sample size: N* = 121  *Age:*  20-40 years: ~ 64%  > 40 years: ~36%  *Gender:*  Male: ~74% Female: ~26% *Working experience:*  < 11 years: ~25% 11-20 years: ~48%  > 20 years: ~27% | Information systems | 51% | Correlation analysis PLS-SEM | - Project performance was significantly associated with leadership, results orientation, emotional intelligence, ethics, creativity, and motivation. - Goal achievement was significantly associated with leadership, emotional intelligence, ethics, and creativity. - User satisfaction was significantly associated with leadership, communication, results orientation, ethics, creativity, and motivation. - Emotional intelligence was a significant predictor of user satisfaction. - Ethics was a significant predictor for goal |
|  |  |  |  |  |  | achievement.   - Creativity and motivation were significant predictors |
|  |  |  |  |  |  | of project performance. |
| 2 | Rana and Shuja [73] | *Sample size: N* = 183  *Age:*  21-30 years: ~52%  31-40 years: ~36%  > 40 years: ~12% | Transportation | NR | SEM | - Leadership competencies, measure as a second-order construct, was not a significant predictor of PS. - Innovative work behavior fully mediates the relationship between leadership competencies and PS. |
|  |  | *Gender:* Not reported |  |  |  |  |
|  |  | *Working experience:* |  |  |  |  |
|  |  | <6: ~48% |  |  |  |  |
|  |  | > 5 years: ~52% |  |  |  |  |
| 3 | Elmezain et al. [74] | *Sample size: N* = 400  *Age:*  < 30 years: ~13% | Construction | 87% | Regression analysis | - Human and political skills were a significant predictor of PS. |
|  |  | 30-49 years: ~52% |  |  |  |  |
|  |  | > 50 years: ~35% |  |  |  |  |
|  |  | *Gender:* |  |  |  |  |
|  |  | Male: ~ 78% |  |  |  |  |
|  |  | Female: ~ 22% |  |  |  |  |
|  |  | *Experience:* |  |  |  |  |
|  |  | < 7 years: ~29% |  |  |  |  |
|  |  | 8-20 years: ~58% |  |  |  |  |
|  |  | >20 years: ~13% |  |  |  |  |
| 4 | Ahmed & Lodhi [75] | *Sample size: N* = 112 *Age*: Not reported *Gender:*  Male: ~ 79% | Information technology and telecommunication public projects | 75% | Correlation analysis Regression analysis | - Self-awareness and resilience were significantly associated with the following PS criteria: organizational success, team satisfaction, user satisfaction, and project performance. |

| **No.** | **Author** | **Sample** | **Sector** | **Response rate** | **Analysis tool** | **Main results** |
| --- | --- | --- | --- | --- | --- | --- |
|  |  | Female: ~ 21%  *Experience:*  < 5 years: ~17% |  |  |  | - Emotional intelligence was a significant predictor of all PS criteria. |
|  |  | 5-10 years: ~59% |  |  |  |  |
|  |  | >15 years: ~23% |  |  |  |  |
| 5 | Irfan et al. [76] | *Sample size: N* = 260 *Age:* Not reported *Gender:* | Public sector organizations | 52% | PLS-SEM | - PMGs’ competencies, measured as a second-order construct, were not a significant predictor of PS. |
|  |  | Male: ~ 78% |  |  |  |  |
|  |  | Female: ~ 22% |  |  |  |  |
|  |  | *Experience:* |  |  |  |  |
|  |  | 5-15 years: ~73% |  |  |  |  |
|  |  | > 15 years: ~27% |  |  |  |  |
| 6 | Lima and Quevedo- Silva [77] | *Sample size*: *N* = 119  *Age:*  mean = 40 years  *Gender:* | Not reported | 88% | PLS-SEM | - Interpersonal skills were a significant predictor of PS. - Emotional intelligence was not a significant predictor of PS. |
|  |  | Male: ~ 81,51% |  |  |  |  |
|  |  | Female: ~ 18,49% |  |  |  |  |
|  |  | *Working experience:* |  |  |  |  |
|  |  | mean = 10 years |  |  |  |  |
| 7 | Khan et al. [78] | *Sample size: N* = 255  *Age:*  25-45 years: ~63% | Construction | NR | PLS-SEM | - Communication and interpersonal skills were a significant predictor for PS. |
|  |  | 46-55 years: ~33% |  |  |  |  |
|  |  | >55 years: ~4% |  |  |  |  |
|  |  | *Gender:* |  |  |  |  |
|  |  | Male: ~ 100% |  |  |  |  |
|  |  | *Experience:* |  |  |  |  |
|  |  | 1-5 years: ~11% |  |  |  |  |
|  |  | 6-10 years: ~55% |  |  |  |  |
|  |  | 11-15 years: ~34% |  |  |  |  |
| 8 | Podgórska and Pichlak [72] | *Sample size: N* = 102  *Age:*  >41 years: ~ 63%  > 40 years: ~37%  *Gender:*  Male: ~ 31%  Female: ~ 69% | Not reported | 58% | Regression analysis | - Conscientiousness was a significant predictor of PS in high-complexity and mandatory projects. - Communication was a significant predictor of PS in medium-complexity projects. - Motivation was a significant predictor of PS in repositioning projects. |
|  |  | *Working experience:* |  |  |  |  |

| **No.** | **Author** | **Sample** | **Sector** | **Response rate** | **Analysis tool** | **Main results** |
| --- | --- | --- | --- | --- | --- | --- |
|  |  | < 6 years: ~64% |  |  |  |  |
|  |  | > 5 years: ~36% |  |  |  |  |
| 9 | Maqbool et al. | *Sample size: N* = 345 | Construction | 84% | Correlation analysis | - Self-awareness and relationship management were |
|  | [15] | *Age:* |  |  | Regression analysis | significantly associated with PS. |
|  |  | 25-45 years: ~ 65% |  |  |  | - Communication reported the highest correlation with |
|  |  | > 45 years: ~35% |  |  |  | PS among all competencies included in the study. |
|  |  | *Gender:* Not reported |  |  |  | - PMGs’ competencies, measured as a second-order |
|  |  | Working experience:  5 to 15 years: ~67% |  |  |  | construct composed by communication, teamwork, attentiveness and managing conflict, were |
|  | | > 15 years: ~33% |  |  |  | significantly associated with PS. |
|  | |  |  |  |  | - Emotional intelligence was a significant predictor of |
|  | |  |  |  |  | PS. |
| 10 Müller and | | *Sample size: N* = 400 | Not reported | Not applicable | Regression analysis | - Strategic perspective was a significant predictor of the |
| Turner [39] | | *Age:* |  | (snowball sampling) |  | following PS criteria: project performance and self- |
|  | | 40-year-old or younger: ~ |  |  |  | defined criteria of success. |
|  | | 27% |  |  |  | - Managing resources perspective was a significant |
|  | | > 40 years: ~73% |  |  |  | predictor for user satisfaction, other stakeholders’ |
|  | | *Gender:* |  |  |  | satisfaction, meeting user requirements, customer |
|  | | Male: ~ 65% |  |  |  | satisfaction and reoccurring business. |
|  | | Female: ~ 34% |  |  |  | - Empowering was a significant predictor for team |
|  | | *Working experience*: Not |  |  |  | satisfaction. |
|  | | reported |  |  |  | - Self-awareness was a significant predictor for project |
|  | |  |  |  |  | achievement. |
|  | |  |  |  |  | - Interpersonal sensitivity was a significant predictor for |
|  | |  |  |  |  | supplier satisfaction. |
|  | |  |  |  |  | - Influence was a significant predictor for other |
|  | |  |  |  |  | stakeholder satisfaction. |
|  | |  |  |  |  | - Motivation was a significant predictor for project |
|  | |  |  |  |  | performance. |
|  | |  |  |  |  | - Conscientiousness was a significant predictor for team |
|  | |  |  |  |  | satisfaction. |

411 Notes. PLS-SEM: Partial Least Squares Structural Equation Modeling, SEM: Structural equation modeling, PS: Project success, PMGs: Project managers, NR: Non-reported.

### 412 5. Discussion

413 413
414 This SLR examined the evidence pertaining to the relationship between PMGs’ competencies and PS. The
415 analysis of the included studies yielded three key findings. Firstly, six distinct clusters of authors were
416 identified, each contributing to the conceptualization and identification of PMGs’ competencies. Secondly,
417 the conceptualization of PS has evolved from a traditional approach centered around criteria such as time,
418 cost, and quality, to a more comprehensive, holistic, and multidimensional perspective. Lastly, through
419 thematic analysis, a total of 12 competencies, organized into four dimensions, were identified as potential
420 determinants of PS. Notably, the most significant competencies associated with PS were found within the
421 personal and social dimensions. A brief discussion of these findings is presented below.
422 422
423 In relation to the first finding, this SLR identified six distinct clusters of authors whose work influenced the
424 competence frameworks utilized in the included articles. These clusters represented conceptualizations
425 proposed by scholars and reputable PM institutions. The in-depth content analysis revealed that the
426 frameworks proposed by Dulewicz and Higgs [79] and Clarke [80] were the most prevalent among the
427 examined articles. Conversely, frameworks developed by Sunindijo [81], Ofori [84], and Nguyen and
428 Hadikusumo [83] were comparatively less frequently employed. Additionally, the PMI [12] emerged as a
429 key institutional point of reference for identifying the competencies required in a PMG. For instance, Lima
430 and Quevedo-Silva [77] and Maqbool et al. [15] studies adopted Clarke’s [80] framework, which was based
431 on the PMI’s [12] (2017b) list of competencies. Elmezain et al. [74], who cited Sunindijo et al. [81] as their
432 framework source, incorporated several competencies defined by the PMI [5], although the majority of these
433 were technical.
434 434
435 Regarding the second finding, the articles examined in this SLR provided support for the view that PS
436 should be understood as a multidimensional construct. This finding aligns with a recent study by Ika and
437 Pinto [54] that revisited the conceptualization of PS. The results of this review indicate that project
438 performance, encompassing time, cost, and quality, emerged as the most commonly considered criterion of
439 success across all the articles. However, a significant number of the included articles also acknowledged
440 additional criteria, leading to the identification of three dimensions of PS. The first dimension refers to the
441 impact on stakeholders and includes criteria related to the satisfaction of various project stakeholders,
442 including clients, users, suppliers, and the project team, among others. The second dimension focuses on
443 the impact of the project on the organization, comprising both short- and long-term improvements. Lastly,
444 the third dimension is related with the general management of the project. This dimension encompasses
445 aspects such as project performance, which includes the traditional "iron triangle" of time, cost, and quality,
446 as well as the achievement of project objectives, adherence to project-defined criteria, and compliance with
447 safety and environmental protocols and regulations. This conceptualization supports the multidimensional
448 nature of PS. However, as noted by Ika and Pinto [54], it is important to highlight that the majority of the
449 included articles overlooked the inclusion of sustainability criteria. Among the entire sample of studies, only
450 one [76] out of ten explicitly addressed compliance with safety and environmental regulations as a criterion
451 of success.
452 452
453 The findings of this SLR have provided insights into the competencies that exhibit a significant
454 relationship with PS. Specifically, the articles included in this review extensively examined competencies
455 associated with the personal and social dimensions, such as leadership, communication, and emotional
456 intelligence. These competencies have been extensively studied in previous literature [18,86], and their
457 impact on PS was explored in the majority of the reviewed articles. Conversely, the influence of other
458 competencies, such as ethics, received less attention and was not extensively explored. Moreover, the
459 empirical evidence gathered in this review suggests that the effect of project management competencies on
460 PS may vary depending on several factors. For instance, the type of project was found to be a significant
461 factor influencing the relationship between competencies and PS [72]. Furthermore, individual and
462 organizational factors were identified as potential mediating variables that could affect the relationship
463 between competencies and PS [73]. These findings highlight the complexity and contextual nature of the
464 relationship between competencies and PS. Next, a brief discussion will be presented to shed light on how
465 these identified competencies can contribute to enhancing PS.
466 466
467 Leadership competence was one of the most studied competencies that improve PS. Although a few of
468 the studies included in this SLR [71,73] reported that it does not have a significant effect on PS, a great
469 number of the studies [15,39,72,74,76] suggested that PMGs’ leadership, conceived as their capacity to
470 influence, empower and develop others, has a positive effect on PS. This finding agrees with the existing
471 literature that has examined its influence on PS [87–90]. The development of competencies such as
472 leadership allows PMGs to motivate their teams to be more productive [91], to show outstanding
473 performance beyond expectations [89], to enhance team cohesion and engagement [92], to foster knowledge
474 transfer across project teams [89], among other positive behaviors that would impact on projects’ outcomes.
475 475
476 The articles included in this SLR demonstrate a significant and positive relationship between
477 communication and PS [15,72,77,78], in agreement with previous research findings [93,94]. The
478 significance of this competence lies in its impact throughout various stages of a project [94]. Effective
479 communication between PMGs and the project team’s members allows better collaboration [95], encourages
480 knowledge sharing [96], and enhances the team’s motivation a sense of inclusivity [94], which contribute
481 to the overall achievement of PS.
482 482
483 The influence of emotional intelligence on PS was assessed in most of the articles included in this SLR.
484 Although some studies reported a non-significant relationship between emotional intelligence [71,73,77],
485 there was evidence supporting a positive association between these two variables [15,39,72,75]. PMGs with
486 high emotional intelligence are more likely to establish stronger relationships with their teams, thereby
487 improving communication, clarity of mission, and support, ultimately enhancing PS [21]. In addition, the
488 development of this competence allows PMGs to adequately regulate their emotions in complex situations,
489 promoting positive behaviors such as empathy, respect, and leadership. These behaviors contribute to their
490 ability to address challenges successfully and ensure higher PS [97,98].
491 491
492 Regarding the influence of PMGs’ ethics, a positive relationship was identified between this competence
493 and PS criteria, particularly goal achievement [71]. Ethics has been acknowledged as a driving force for the
494 advancement of the PM profession [48] and an essential competence that PMGs should possess [99,100].
495 However, empirical evidence on the impact of ethics on PS remains limited. Some related terms, such as
496 honesty, integrity, and transparency [71,74], or ethical thinking [100], ethical decision-making, and ethics
497 sensitivity have been addressed in previous studies. However, its effect on PS has rarely been estimated and
498 reported. The evidence found on ethics in this SLR was obtained from information systems and construction
499 projects. Future studies could explore the influence of this competence in different industries and countries.
500 500

### 501 6. Limitations and strengths

502 502
503 While this review contributes with some insights to the PM literature, it is important to mention its
504 limitations. Firstly, the time frame of the review from 2010 to 2022 may have resulted in the exclusion of
505 relevant articles that explore the relationship between PMGs’ competencies and PS. It is possible that some
506 studies conducted outside this timeframe may provide further insights into the topic. Secondly, the use of
507 specific search terms such as “competence,” “competency,” “competences,” “competencies,” “skill,” and
508 “skills” may have excluded other studies [86,88] that examined the impact of different competencies
509 individually. Including an exhaustive list of competencies in the search strings could have introduced
510 significant heterogeneity into the reviewed articles, potentially limiting the ability to provide a
511 comprehensive review of the existing literature.
512 512
513 Despite these limitations, this SLR makes several notable contributions to the PM discipline. First, it fills a
514 gap in the existing literature by synthesizing available empirical evidence on the relationship between
515 PMGs’ competencies and PS. Second, the review conducts a thematic analysis and adopts a holistic
516 perspective to categorize the PMGs’ competencies that are associated with PS. Third, this review highlights
517 the primary authors and PM institutions that have significantly influenced the conceptualization of PMGs’
518 competencies. Four, the review examines the criteria used to measure PS in the included articles and
519 organizes them into three dimensions, offering a nuanced understanding of the multifaceted nature of PS
520 measurement.
521 521

### 522 7. Conclusions

523 523
524 The present SLR extends the literature in project management concerning the influence of PMGs’
525 competencies on PS. Despite the growing interest in addressing the role of PMGs’ competencies to achieve
526 higher success, to the best of our knowledge, there is a lack of systematic reviews that present an analysis
527 of the available evidence on the relationship between PMGs’ competencies on PS. To fill this gap in the
528 literature, this SLR analyzed the existing evidence regarding this relationship. Three main conclusions can
529 be derived from the findings of this review. First, the existing literature has primarily explored the influence
530 on PS of PMGs’ competencies from the personal and social dimensions, such as leadership, communication,
531 and emotional intelligence. Second, PS is a multidimensional construct that comprises three main
532 dimensions: impact on stakeholders, impact on the organization, and general project management. Third,
533 the available data suggested that greater levels of PMGs’ competencies are associated with improved PS.
534 These findings may support scholars and managers to understand the mechanisms through which individual
535 characteristics, such as competencies, may allow PMGs to achieve better outcomes.
536 536
537 This SLR contributes to the existing literature in the PM discipline by offering a comprehensive synthesis
538 of empirical evidence, providing a thorough overview of the current state of knowledge regarding the
539 relationship between PMGs’ competencies and PS. In addition, this SLR identifies key contributors and
540 sources of knowledge in the field, offering a valuable reference point for further research and exploration.
541 The study also offers a review on how PS is conceptualized and measured. Moreover, it presents a
542 classification of PMGs’ competencies that influence PS. Through a thematic analysis of the competencies
543 examined in the included articles, this categorization provides valuable insights into the emphasis placed on
544 different types of competencies. It highlights the significant attention given to personal and social
545 competencies, while pointing out the relatively limited exploration of sustainability, cultural, or digital
546 competencies [85].
547 547
548 **Funding:** This study did not receive any specific grant from funding agencies in the public, commercial, or
549 not-for-profit sectors.
550 550
551 **Conflict of Interest Statement:** The authors declare no conflict of interest.
552 552
553 **Data Availability Statement:** All files are available either within the manuscript (Tables 1, 2, 3, 4) or as
554 supplementary files. Hyperlinks are provided within the manuscript after the reference list.
555 555
556 556

### 557 References

558 558
559 [1]	Martinsuo M, Teerikangas S, Stensaker I, Meredith J. Managing strategic projects and programs in
560 and between organizations. Int J Proj Manag. 2022;40: 499–504.

561	doi:10.1016/j.ijproman.2022.06.003

562	[2]	Wu T. Digital project management: rapid changes define new working environments. J Bus

563	Strategy. 2022;43: 323–331. doi:10.1108/JBS-03-2021-0047

564	[3]	Schoemaker PJH, Heaton S, Teece D. Innovation, Dynamic Capabilities, and Leadership. Calif

565	Manage Rev. 2018;61: 15–42. doi:10.1177/0008125618790246

566	[4]	Martinsuo M, Ahola T. Multi-project management in inter-organizational contexts. Int J Proj

567	Manag. 2022;40: 813–826. doi:10.1016/j.ijproman.2022.09.003

568	[5]	Project Management Institute. Project Management Job Growth and Talent Gap 2017–2027. 2017

569	May [cited 2023 April 15]. Available from: [https://www.pmi.org/-](https://www.pmi.org/-/media/pmi/documents/public/pdf/learning/job-growth-report.pdf)

570	[/media/pmi/documents/public/pdf/learning/job-growth-report.pdf](https://www.pmi.org/-/media/pmi/documents/public/pdf/learning/job-growth-report.pdf)

571	[6]	Crawford L, Nahmias AH. Competencies for managing change. Int J Proj Manag. 2010;28: 405–

572	412. doi:10.1016/j.ijproman.2010.01.015

573	[7]	Ahsan K, Ho M, Khan S. Recruiting Project Managers: A Comparative Analysis of Competencies

574	and Recruitment Signals from Job Advertisements. Proj Manag J. 2013;44: 36–54.

575	doi:10.1002/pmj.21366

576	[8]	Moradi S, Kähkönen K, Aaltonen K. Comparison of research and industry views on project

577	managers’ competencies. Int J Manag Proj Bus. 2020;13: 543–572. doi:10.1108/IJMPB-04-2019-

578	0085

579	[9]	Moradi S, Kähkönen K, Aaltonen K. Project Managers’ Competencies in Collaborative

580	Construction Projects. Buildings. 2020;10: 50. doi:10.3390/buildings10030050

581	[10] Chen T, Fu M, Liu R, Xu X, Zhou S, Liu B. How do project management competencies change

582	within the project management career model in large Chinese construction companies? Int J Proj

583	Manag. 2019;37: 485–500. doi:10.1016/j.ijproman.2018.12.002

584	[11] Ribeiro A, Amaral A, Barros T. Project Manager Competencies in the context of the Industry 4.0.

585	Procedia Comput Sci. 2021;181: 803–810. doi:10.1016/j.procs.2021.01.233

586 [12] Project Management Institute. Project Manager Competency Development Framework. 3rd ed.
587 Newtown Square (PA): Project Management Institute, Inc.; 2017.
588 [13] International Project Management Association. Individual Competence Baseline for Project,
589 Programme and Portfolio Management (Version 4.0.). Zurich: International Project Management
590 Association; 2015.
591 [14] Monteiro de Carvalho M, Rabechini JrR. Impact of risk management on project performance: the

592	importance of soft skills. Int J Prod Res. 2015;53: 321–340. doi:10.1080/00207543.2014.919423

593	[15] Maqbool R, Sudong Y, Manzoor N, Rashid Y. The Impact of Emotional Intelligence, Project

594	Managers’ Competencies, and Transformational Leadership on Project Success: An Empirical

595	Perspective. Proj Manag J. 2017;48: 58–75. doi:10.1177/875697281704800304

596	[16] Gruden N, Stare A. The Influence of Behavioral Competencies on Project Performance. Proj Manag

597	J. 2018;49: 98–109. doi:10.1177/8756972818770841

598 [17] Zaman U, Jabbar Z, Nawaz S, Abbas M. Understanding the soft side of software projects: An
599 empirical study on the interactive effects of social skills and political skills on complexity –
600 performance relationship. Int J Proj Manag. 2019;37: 444–460. doi:10.1016/j.ijproman.2019.01.015
601 [18] Alvarenga JC, Branco RR, Guedes ALA, Soares CAP, Silva WdSe. The project manager core
602 competencies to project success. Int J Manag Proj Bus. 2020;13: 277–292. doi:10.1108/IJMPB-12-

603	2018-0274

604	[19] Abbas M, Ali R. Transformational versus transactional leadership styles and project success: A

605	meta-analytic review. Eur Manag J. 2023;41: 125–142. doi:10.1016/j.emj.2021.10.011

606	[20] Castro M, Barcaui A, Bahli B, Figueiredo R. Do the Project Manager’s Soft Skills Matter? Impacts

607	of the Project Manager’s Emotional Intelligence, Trustworthiness, and Job Satisfaction on Project

608	Success. Adm Sci. 2022;12: 141. doi:10.3390/admsci12040141

609	[21] Rezvani A, Chang A, Wiewiora A, Ashkanasy NM, Jordan PJ, Zolin R. Manager emotional

610	intelligence and project success: The mediating role of job satisfaction and trust. Int J Proj Manag.

611	2016;34: 1112–1122. doi:10.1016/j.ijproman.2016.05.012

612	[22] McKevitt D, Carbery R, Collins S. Job crafting in project management: Implications for project

613	success and career satisfaction. Int J Proj Manag. 2022;40: 741–749.

614	doi:10.1016/j.ijproman.2022.08.004

615	[23] Cao J, Liu C, Zhou Y, Duan K. Work-to-Family Conflict, Job Burnout, and Project Success among

616	Construction Professionals: The Moderating Role of Affective Commitment. Int J Environ Res

617	Public Health. 2020;17: 2902. doi:10.3390/ijerph17082902

618	[24] Sarwar A, Imran MK, Anjum Z-U-Z, Zahid U. How innovative climate leads to project success: the

619	moderating role of gender and work culture. Innov Manag Rev. 2020;17: 413–430.

620	doi:10.1108/INMR-08-2019-0096

621	[25] Adekiya A, Bernardes OTF. Effect of organisational culture on ICT project success in public higher

622	learning institutions in Nigeria. Int J Crit Infrastruct. 2020;16: 187–210.

623	doi:10.1504/IJCIS.2020.108472

624	[26] Ludviga I, Sluka I. Cultural Diversity in Project Management: How Project Success is Perceived in

625	Different Cultures. Int J Organ Divers. 2018;18: 1–12. doi:10.18848/2328-6261/CGP/v18i01/1-12

626	[27] Young R, Chen W, Quazi A, Parry W, Wong A, Poon SK. The relationship between project

627	governance mechanisms and project success: An international data set. Int J Manag Proj Bus.

628	2020;13: 1496–1521. doi:10.1108/IJMPB-10-2018-0212

629	[28]	Imam H. Roles of Shared Leadership, Autonomy, and Knowledge Sharing in Construction Project

630	Success. J Constr Eng Manag. 2021;147: 04021067. doi:10.1061/(ASCE)CO.1943-7862.0002084

631	[29] Imam H, Kashif M. Shared leadership and project success: The roles of knowledge sharing,

632	cohesion and trust in the team. Int J Proj Manag. 2021;39: 463–473.

633	doi:10.1016/j.ijproman.2021.02.006

634	[30] Khalifeh A, Farrell P, Al-edenat M. The impact of project sustainability management (PSM) on

635	project success: A systematic literature review. J Manag Dev. 2020;39: 453–474. doi:10.1108/JMD-

636	02-2019-0045

637	[31] Ahmed R, Philbin SP, Cheema F-A. Systematic literature review of project manager’s leadership

638	competencies. Eng Constr Archit Manag. 2021;28: 1–30. doi:10.1108/ECAM-05-2019-0276

639	[32] Pariafsai F, Behzadan AH. Core Competencies for Construction Project Management: Literature

640	Review and Content Analysis. J Civ Eng Educ. 2021;147: 04021010. doi:10.1061/(ASCE)EI.2643-

641	9115.0000051

642	[33] Iriarte C, Bayona Orè S. Soft Skills for IT Project Success: A Systematic Literature Review. In:

643	Mejia J, Muñoz M, Rocha Á, Quiñonez Y, Calvo-Manzano J, editors. Trends and Applications in

644	Software Engineering. Cham: Springer International Publishing; 2018. pp. 147–158.

645	doi:10.1007/978-3-319-69341-5_14

646	[34] Goleman D, Boyatzis R, McKee A. Primal Leadership: Unleashing the Power of Emotional

647	Intelligence. Boston: Harvard Business Press; 2013.

648	[35] Boyatzis R. The Competent Manager: A Model for Effective Performance. New York: John Wiley

649	& Sons; 1982.

650	[36] McClelland DC. Testing for competence rather than for “intelligence.” Am Psychol. 1973;28: 1–14.

651	doi:10.1037/h0034092

652	[37] Loufrani-Fedida S, Missonier S. The project manager cannot be a hero anymore! Understanding

653	critical competencies in project-based organizations from a multilevel approach. Int J Proj Manag.

654	2015;33: 1220–1235. doi:10.1016/j.ijproman.2015.02.010

655	[38] Boyatzis RE. Competencies in the 21st century. J Manag Dev. 2008;27: 5–12.

656	doi:10.1108/02621710810840730

657	[39] Müller R, Turner R. Attitudes and leadership competences for project success. Balt J Manag.

658	2010;5: 307–329. doi:10.1108/17465261011079730

659	[40] Nahod M-M, Radujković MVM. The Impact of ICB 3.0 Competences on Project Management

660	Success. Procedia - Soc Behav Sci. 2013;74: 244–254. doi:10.1016/j.sbspro.2013.03.014

661	[41] Lambert DSL, Monteiro de Carvalho M. The influence of project leaders’ behavioral competencies

662	on the performance of Six Sigma projects. Rev Bus Manag. 2016;18: 609–632.

663	doi:10.7819/rbgn.v18i62.2450

664	[42] Müller R, Turner R. Leadership competency profiles of successful project managers. Int J Proj

665	Manag. 2010;28: 437–448. doi:10.1016/j.ijproman.2009.09.003

666	[43] Hanna AS, Ibrahim MW, Lotfallah W, Iskandar KA, Russell JS. Modeling Project Manager

667	Competency: An Integrated Mathematical Approach. J Constr Eng Manag. 2016;142: 04016029.

668	doi:10.1061/(ASCE)CO.1943-7862.0001141

669	[44] Bashir R, Sajjad A, Bashir S, Latif KF, Attiq S. Project Managers’ Competencies in International

670	Development Projects: A Delphi Study. SAGE Open. 2021;11: 21582440211058188.

671	doi:10.1177/21582440211058188

672	[45] Saunders JW, Nunes B, Monteiro de Carvalho M. Project Managers’ Competences: What Do Job

673	Advertisements and the Academic Literature Say? Proj Manag J. 2018;49: 82–97.

674	doi:10.1177/8756972818770884

675	[46] Khattak MS, Mustafa U. Management competencies, complexities and performance in engineering

676	infrastructure projects of Pakistan. Eng Constr Archit Manag. 2019;26: 1321–1347.

677	doi:10.1108/ECAM-05-2017-0079

678	[47]	Khattak MS, Mustafa U, Rehman AU, Khattak IU. Impact	of	Management	Competencies and

679	Complexities	on Performance	in	Public	Sector	Infrastructure	Projects	of Pakistan. NUML

680	Int J Bus Manag. 2016;11: 12–34.

681	[48] Project Management Institute. Pulse of the Profession® 2023: Power Skills, Redefining Project

682	Success. 2023 [cited 2023 April 15]. Available from: [https://www.pmi.org/learning/thought-](https://www.pmi.org/learning/thought-leadership/pulse/power-skills-redefining-project-success)

683	[leadership/pulse/power-skills-redefining-project-success](https://www.pmi.org/learning/thought-leadership/pulse/power-skills-redefining-project-success)

684	[49] Crawford L. Senior management perceptions of project management competence. Int J Proj Manag.

685	2005;23: 7–16. doi:10.1016/j.ijproman.2004.06.005

686	[50] Pinto JK, Davis K, Ika LA, Jugdev K, Zwikael O. Coming to terms with project success: Current

687	perspectives and future challenges. Int J Proj Manag. 2022;40: 831–834.

688	doi:10.1016/j.ijproman.2022.09.001

689	[51] Cserháti G, Szabó L. The relationship between success criteria and success factors in organisational

690	event projects. Int J Proj Manag. 2014;32: 613–624. doi:10.1016/j.ijproman.2013.08.008

691	[52] Ika LA. Project success as a topic in project management journals. Proj Manag J. 2009;40: 6–19.

692	doi:10.1002/pmj.20137

693	[53] Westerveld E. The Project Excellence Model®: linking success criteria and critical success factors.

694	Int J Proj Manag. 2003;21: 411–418. doi:10.1016/S0263-7863(02)00112-6

695	[54] Ika LA, Pinto JK. The “re-meaning” of project success: Updating and recalibrating for a modern

696	project management. Int J Proj Manag. 2022;40: 835–848. doi:10.1016/j.ijproman.2022.08.001

697	[55] De Wit A. Measurement of project success. Int J Proj Manag. 1988;6: 164–170. doi:10.1016/0263-

698	7863(88)90043-9

699	[56] Shenhar AJ, Dvir D, Levy O, Maltz AC. Project Success: A Multidimensional Strategic Concept.

700	Long Range Plann. 2001;34: 699–725. doi:10.1016/S0024-6301(01)00097-8

701	[57] McLeod L, Doolin B, MacDonell SG. A Perspective-Based Understanding of Project Success. Proj

702	Manag J. 2012;43: 68–86. doi:10.1002/pmj.21290

703	[58] Müller R, Turner R. The Influence of Project Managers on Project Success Criteria and Project

704	Success by Type of Project. Eur Manag J. 2007;25: 298–309. doi:10.1016/j.emj.2007.06.003

705	[59]	Monteiro de Carvalho M, Rabechini JrR. Can project sustainability management impact project

706	success? An empirical study applying a contingent approach. Int J Proj Manag. 2017;35: 1120–

707	1132. doi:10.1016/j.ijproman.2017.02.018

708	[60] Shenhar AJ, Dvir D. Reinventing project management: the diamond approach to successful growth

709	and innovation. Boston: Harvard Business Review Press; 2007.

710	[61] Zwikael O, Meredith J. Evaluating the Success of a Project and the Performance of Its Leaders.

711	IEEE Trans Eng Manag. 2021;68: 1745–1757. doi:10.1109/TEM.2019.2925057

712	[62] Atkinson R. Project management: cost, time and quality, two best guesses and a phenomenon, its

713	time to accept other success criteria. Int J Proj Manag. 1999;17: 337–342. doi:10.1016/S0263-

714	7863(98)00069-6

715	[63] Jugdev K, Müller R. A Retrospective look at our Evolving Understanding of Project Success. Proj

716	Manag J. 2005;36: 19–31. doi:10.1177/875697280503600403

717	[64] Serrador P, Turner R. The Relationship between Project Success and Project Efficiency. Proj

718	Manag J. 2015;46: 30–39. doi:10.1002/pmj.21468

719	[65] Crawford L. Measuring Performance. In: Turner R, editor. Gower Handbook of Project

720	Management. 5th ed. New York: Routledge; 2014. pp. 105–120.

721	[66] Andersen ES, Birchall D, Arne Jessen S, Money AH. Exploring project success. Balt J Manag.

722	2006;1: 127–147. doi:10.1108/17465260610663854

723	[67] Zhao Y, Pinto Llorente AM, Sánchez Gómez MC. Digital competence in higher education research:

724	A systematic literature review. Comput Educ. 2021;168: 104212.

725	doi:10.1016/j.compedu.2021.104212

726	[68] Nowell LS, Norris JM, White DE, Moules NJ. Thematic Analysis: Striving to Meet the

727	Trustworthiness Criteria. Int J Qual Methods. 2017;16: 1609406917733847.

728	doi:10.1177/1609406917733847

729	[69] McLaren CD, Bruner MW. Citation network analysis. Int Rev Sport Exerc Psychol. 2022;15: 179–

730	198. doi:10.1080/1750984X.2021.1989705

731	[70] Martinez-Perez C, Alvarez-Peregrina C, Villa-Collar C, Sánchez-Tena MÁ. Current State and

732	Future Trends: A Citation Network Analysis of the Academic Performance Field. Int J Environ Res

733	Public Health. 2020;17: 5352. doi:10.3390/ijerph17155352

734	[71] Sampaio S, Wu Q, Cormican K, Varajão J. Reach for the sky: analysis of behavioral competencies

735	linked to project success. Int J Manag Proj Bus. 2021;15: 192–215. doi:10.1108/IJMPB-09-2020-

736	0276

737	[72] Podgórska M, Pichlak M. Analysis of project managers’ leadership competencies: Project success

738	relation: what are the competencies of polish project leaders? Int J Manag Proj Bus. 2019;12: 869–

739	887. doi:10.1108/IJMPB-08-2018-0149

740	[73] Rana A, Shuja A. Influence of Leadership Competencies on Transport Infrastructure Projects’

741	Success: A Mediated Moderation through Innovative-Work-Behavior and the Project Type. Pak J

742	Commer Soc Sci. 2022;16: 1–33.

743	[74] Elmezain M, Baduruzzaman WHW, Khoiry MA. The Impact of Project Manager’s Skills and Age

744	on Project Success. Braz J Oper Prod Manag. 2021;18: 1–16. doi:10.14488/BJOPM.2021.017

745	[75] Ahmed R, Lodhi KM. Do Project Managers’ Emotional Leadership Competencies Affect the

746	Success of Public Sector Projects in Pakistan? Int J Inf Technol Proj Manag. 2021;12: 83–98.

747	doi:10.4018/IJITPM.2021040105

748	[76] Irfan M, Khan SZ, Hassan N, Hassan M, Habib M, Khan S, et al. Role of Project Planning and

749	Project Manager Competencies on Public Sector Project Success. Sustainability. 2021;13: 1421.

750	doi:10.3390/su13031421

751	[77] Lima LF, Quevedo-Silva F. Emotional intelligence and success of project management: the

752	mediating effect of interpersonal skills. Int J Proj Organ Manag. 2020;12: 54–73.

753	doi:10.1504/IJPOM.2020.105714

754	[78] Khan R, Huang J, Abas M, Iqbal Z. Role of Project Manager’s Competencies towards Project

755	Success: An Empirical Evidence from Public Art and Construction Industry of Pakistan. Pac Bus

756	Rev Int. 2020;12: 51–62.

757	[79] Dulewicz V, Higgs M. Assessing leadership styles and organisational context. J Manag Psychol.

758	2005;20: 105–123. doi:10.1108/02683940510579759

759	[80] Clarke N. Emotional Intelligence and Its Relationship to Transformational Leadership and Key

760	Project Manager Competences. Proj Manag J. 2010;41: 5–20. doi:10.1002/pmj.20162

761	[81] Sunindijo RY. Project manager skills for improving project performance. Int J Bus Perform Manag.

762	2015;16: 67–83. doi:10.1504/IJBPM.2015.066041

763	[82] Katz RL. Skills of an Effective Administrator. Harv Bus Rev. 1974;52: 90–102.

764	[83] Nguyen HT, Hadikusumo B. Impacts of human resource development on engineering, procurement,

765	and construction project success. Built Environ Proj Asset Manag. 2017;7: 73–85.

766	doi:10.1108/BEPAM-04-2016-0010

767	[84] Ofori D. An Exploratory Study of Project Management Competency in Ghana. J Afr Bus. 2014;15:

768	197–210. doi:10.1080/15228916.2014.956644

769	[85]	Ochoa P, Jáuregui K, Gomes T, Ruiz B, Lasio V. Las competencias laborales en el mercado de

770	profesionales de América Latina: ¿Qué destrezas demandarán las empresas del futuro en Chile,

771	Colombia, Ecuador y el Perú? Lima: ESAN Ediciones; 2018.

772	[86] Cleveland S, Cleveland M. Leadership Competencies for Sustained Project Success. Int J Appl

773	Manag Theory Res. 2020;2: 35–47. doi:10.4018/IJAMTR.2020010103

774	[87] Khan J, Jaafar M, Javed B, Mubarak N, Saudagar T. Does inclusive leadership affect project

775	success? The mediating role of perceived psychological empowerment and psychological safety. Int

776	J Manag Proj Bus. 2020;13: 1077–1096. doi:10.1108/IJMPB-10-2019-0267

777	[88] Yang L-R, Huang C-F, Wu K-S. The association among project manager’s leadership style,

778	teamwork and project success. Int J Proj Manag. 2011;29: 258–267.

779	doi:10.1016/j.ijproman.2010.03.006

780	[89] Aga DA, Noorderhaven N, Vallejo B. Transformational leadership and project success: The

781	mediating role of team-building. Int J Proj Manag. 2016;34: 806–818.

782	doi:10.1016/j.ijproman.2016.02.012

783	[90] Mariam S, Khawaja KF, Qaisar MN, Ahmad F. Knowledge-Oriented Leadership, Team Cohesion,

784	and Project Success: A Conditional Mechanism. Proj Manag J. 2022;53: 128–145.

785	doi:10.1177/87569728211063128

786	[91] Fareed MZ, Su Q, Almutairi M, Munir K, Fareed MMS. Transformational leadership and project

787	success: The mediating role of trust and job satisfaction. Front Psychol. 2022;13: 954952.

788	doi:10.3389/fpsyg.2022.954052

789	[92] Raziq MM, Borini FM, Malik OF, Ahmad M, Shabaz M. Leadership styles, goal clarity, and project

790	success: Evidence from project-based organizations in Pakistan. Leadersh Organ Dev J. 2018;39:

791	309–323. doi:10.1108/LODJ-07-2017-0212

792	[93] Majeed H, Kayani UN, Haider SA. The Project Communication and Trust Nexus as an Antecedents

793	of Project Success: Moderating Role of Authentic Leadership. Int J Bus Commun. 2021;

794	Forthcoming. doi:10.1177/23294884211019098

795	[94] Salman A, Jaafar M, Malik S, Mohammad D, Muhammad SA. An Empirical Investigation of the

796	Impact of the Communication and Employee Motivation on the Project Success Using Agile

797	Framework and Its Effect on the Software Development Business. Bus Perspect Res. 2021;9: 46–

798	61. doi:10.1177/2278533720902915

799	[95] Taleb H, Ismail S, Wahab MH, Mardiah WN, Rani WM, Amat RC. An Overview of Project

800	Communication Management in Construction Industry Projects. J Manag Econ Ind Organ. 2017; 1–

801	9. doi:10.31039/jomeino.2017.1.1.1

802	[96] Pollack J, Matous P. Testing the impact of targeted team building on project team communication

803	using social network analysis. Int J Proj Manag. 2019;37: 473–484.

804	doi:10.1016/j.ijproman.2019.02.005

805	[97] Fareed MZ, Su Q, Awan AA. The effect of emotional intelligence, intellectual intelligence and

806	transformational leadership on project success; an empirical study of public projects of Pakistan.

807	Proj Leadersh Soc. 2021;2: 100036. doi:10.1016/j.plas.2021.100036

808	[98] Zhang Q, Hao S. Developing a mechanism of construction project manager’s emotional intelligence

809	on project success: A grounded theory research based in China. Front Psychol. 2022;13: 693516.

810	doi:10.3389/fpsyg.2022.693516

811	[99] Brière S, Proulx D, Flores ON, Laporte M. Competencies of project managers in international

812	NGOs: Perceptions of practitioners. Int J Proj Manag. 2015;33: 116–125.

813	doi:10.1016/j.ijproman.2014.04.010

814	[100] Helgadóttir H. The ethical dimension of project management. Int J Proj Manag. 2008;26: 743–748.

815	doi:10.1016/j.ijproman.2007.11.002

Figure 1 (PDF format)	[Click here to access/download;Figure;prisma.pdf](https://www.editorialmanager.com/pone/download.aspx?id=33707676&guid=56c0ad36-e114-402b-a78c-9c210ca93102&scheme=1)

Included

Screening

Identification

Records identified through database searching (n = 232)

Web of Science (n = 25) Scopus (n = 53) ScienceDirect (n = 6) ProQuest (n = 148)

Duplicates removed (n = 60)

## ~~>~~

Records after removing duplicates

(n = 172)

Records removed when applying inclusion and exclusion criteria (n = 131)

Figure 1 (EPS format)

p

Articles excluded based on title (n = 7)

Total articles assessed for eligibility (n = 41)

p

Articles excluded based on abstract (n = 8)

Abstracts retrieved

(n = 34)

p

Articles excluded based on full text

(n = 13)

Full-text articles retrieved

(n = 26)

Click here to access/download;Figure;prisma.eps

p

Articles excluded based on quality

(n = 3)

Studies included in quality appraisal

(n = 13)

Studies included in the review (n = 10)

Figure 2 (PDF format)	[Click here to access/download;Figure;citation_network.pdf](https://www.editorialmanager.com/pone/download.aspx?id=33707698&guid=5dc60dbf-1b66-42f9-90ea-7b4a42646276&scheme=1)

Maker and

Zhang and Fan {2013}

Phefwani (2003)

Barry and Plessis (2007)

Ahmed and Lodhi

M iilier and Turner (2007b)

Adams and Anantatm ula (2010) •

Sunindjo et a/. (2017)

ma

Ali and Chileshe (2009)

Druskat and Druskat (2006, 2012)

Clarke (2010)

and Peterson (2004)

(1974)

et a/. (2008)

Farroqui era/. (2008)

Maqbool et *al.* (2017)

|Pu^(zooO,3o12,zois)

inty et a/. (2005a)

Hawkins and Oulewicz (2007)

M tiller and Turner (2010c)

Muller and Turner (2010a)

Click here to access/download;Figure;citation_network.eps

Khan and Sukhotu

*al.* (2021)

etaf. (2006)

Figure 2 (EPS format)

S1. PRISMA Checklist

# [Click here to access/download](https://www.editorialmanager.com/pone/download.aspx?id=33739413&guid=30f004e7-6808-4875-a0e5-c04ea09fabff&scheme=1)

[**Supporting Information** PRISMA_2020_checklist_rev.docx](https://www.editorialmanager.com/pone/download.aspx?id=33739413&guid=30f004e7-6808-4875-a0e5-c04ea09fabff&scheme=1)

S2. Quality assessment criteria

# [Click here to access/download](https://www.editorialmanager.com/pone/download.aspx?id=33739415&guid=2f4c1b9c-ca47-4469-b020-14cfdf45338d&scheme=1)

[**Supporting Information** qualit_criteria.pdf](https://www.editorialmanager.com/pone/download.aspx?id=33739415&guid=2f4c1b9c-ca47-4469-b020-14cfdf45338d&scheme=1)

S3. Quality assessment results

# [Click here to access/download](https://www.editorialmanager.com/pone/download.aspx?id=33739417&guid=5f9de300-3bd4-4c52-94ae-df37cbc7cab6&scheme=1)

[**Supporting Information** matriz_calidad.pdf](https://www.editorialmanager.com/pone/download.aspx?id=33739417&guid=5f9de300-3bd4-4c52-94ae-df37cbc7cab6&scheme=1)
